# Design, synthesis, and *in vitro* evaluation of aza-peptide aldehydes and ketones as novel and selective protease inhibitors

**DOI:** 10.1080/14756366.2020.1781107

**Published:** 2020-07-07

**Authors:** Thomas S. Corrigan, Leilani M. Lotti Diaz, Sarah E. Border, Steven C. Ratigan, Kayla Q. Kasper, Daniel Sojka, Pavla Fajtova, Conor R. Caffrey, Guy S. Salvesen, Craig A. McElroy, Christopher M. Hadad, Özlem Doğan Ekici

**Affiliations:** a Department of Chemistry and Biochemistry, The Ohio State University, Columbus, OH, USA; b Division of Medicinal Chemistry and Pharmacognosy, College of Pharmacy, The Ohio State University, Columbus, OH, USA; c Institute of Parasitology, Biology Centre of the Czech Academy of Sciences, Ceske Budejovice, Czech Republic; d Center for Discovery and Innovation in Parasitic Diseases, Skaggs School of Pharmacy and Pharmaceutical Sciences, University of California San Diego, La Jolla, CA, USA; e Sanford Burnham Prebys Medical Discovery Institute, La Jolla, CA, USA; f Department of Chemistry and Biochemistry, The Ohio State University at Newark, Newark, OH, USA

**Keywords:** Proteasome inhibitor, caspase and legumain inhibitors, aza-peptide carbonyls, anticancer, antiparasitic

## Abstract

Aza-peptide aldehydes and ketones are a new class of reversible protease inhibitors that are specific for the proteasome and clan CD cysteine proteases. We designed and synthesised aza-Leu derivatives that were specific for the chymotrypsin-like active site of the proteasome, aza-Asp derivatives that were effective inhibitors of caspases-3 and -6, and aza-Asn derivatives that inhibited *S. mansoni* and *I. ricinus* legumains. The crystal structure of caspase-3 in complex with our caspase-specific aza-peptide methyl ketone inhibitor with an aza-Asp residue at P1 revealed a covalent linkage between the inhibitor carbonyl carbon and the active site cysteinyl sulphur. Aza-peptide aldehydes and ketones showed no cross-reactivity towards cathepsin B or chymotrypsin. The initial *in vitro* selectivity of these inhibitors makes them suitable candidates for further development into therapeutic agents to potentially treat multiple myeloma, neurodegenerative diseases, and parasitic infections.

## Introduction

**Figure 1.**
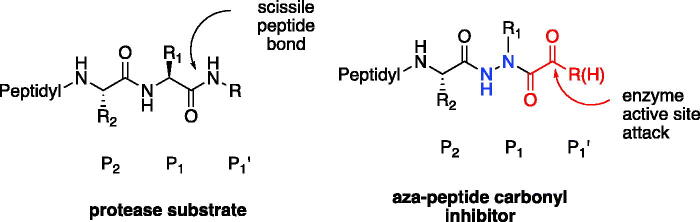
Aza-peptide aldehyde and ketone design.

Aza-peptides are peptidomimetics where the α-carbon centre of one or more of the natural amino acids in a peptide chain is replaced with a nitrogen atom (Figure 1). This modification results in the loss of chirality of the amino acid and a bend in the peptide chain. Previously, it has been shown that the aza-substitution in biologically active peptide analogs has led to enhanced metabolic stability and selectivity for the biological targets^1^. Hence, in medicinal chemistry, aza-peptides have been employed in many roles including enzyme inhibitors, activity-based probes, ligands for receptors, imaging agents, pro-drugs, and drugs. While studies on retro hydrazino-aza-peptoids have been attempted with the proteasome before^2^, aza-peptide aldehydes and ketones emerge as a new class of electrophilic protease inhibitors for multiple proteases. Here we report the design, synthesis, and kinetic evaluation of aza-peptide aldehydes and ketones that are specific for the proteasome, caspases, and legumain.

Incorporation of the aza-peptide motif in protease inhibition was first introduced by Dolle and co-workers^3^. Several aza-peptidyl inhibitors, including aza-peptide halomethyl ketones and aza-peptide ketones, were designed and synthesised to target the clan CA cysteine proteases cathepsin B and calpain, as well as the serine proteases chymotrypsin and elastase. All of them were found to be ineffective inhibitors of these enzymes. However, to date, several other classes of aza-peptidyl inhibitors have been reported as potent and selective inhibitors of other proteases. Aza-peptide epoxides and aza-peptide Michael acceptors have been shown to potently and selectively inhibit clan CD proteases such as caspases, legumains, gingipains, and clostripain by Powers and co-workers^4–7^. Aza-peptide nitriles were developed as effective and stable inhibitors for clan CA proteases such as cathepsins L, S, and K by Guetschow^8^^,^^9^.

In our design, the α-carbon of the P1 amino acid is replaced with a nitrogen, making it an aza-P_1_ residue (Figure 1). A carbonyl group is attached to the P_1_ aza-amino acid replacing the scissile bond of the natural substrate and creating the novel aza-peptide aldehyde or ketone warhead.

The proteasome is a threonine protease and plays a central role in the ubiquitin-mediated degradation of unwanted proteins in the cell^10^. The proteasome is 750 kDa in molecular mass and has three active sites within its 20S catalytic core: chymotrypsin-like (CT-L), trypsin-like (T-L), and caspase-like (C-L)^11^. Inhibition of the proteasome, which has been shown to be overexpressed in tumour cells^12^, leads to cellular apoptosis of the affected cells. Therefore, this inhibition strategy has been validated as an effective treatment for multiple myeloma patients^13^. Inhibitors, such as the aza-peptide aldehydes and ketones described here, are designed to cease proteolysis of mis-folded and unwanted proteins through the selective inhibition of the CT-L active site. Development of the proteasome inhibitor blockbuster drugs bortezomib (FDA-approved in 2003), carfilzomib^14^^,^^15^ (FDA-approved in 2012) and ixazomib (FDA-approved in 2015) revolutionised the treatment of multiple myeloma, significantly prolonging the lives of patients^16^. However, bortezomib shows severe side effects, such as peripheral neuropathy, in up to 64% of newly diagnosed multiple myeloma patients,^17^^,^^18^ largely due to off-target inhibition, by bortezomib’s boronic acid warhead^19^, of HtrA2/Omi, a serine protease involved in neuronal survival. Carfilzomib has been shown to result in less peripheral neuropathy, but lately it has been associated with adverse cardiovascular effects in up to 30% of the treated patients^20^. Ixazomib is an oral formulation and also possesses a boronic acid warhead; thus, peripheral neuropathy is still a major side effect. Therefore, careful dosing or discontinuation is also essential with ixazomib, as drug-induced peripheral neuropathy can be irreversible^21^. Hence, the search for new proteasome inhibitors is an ongoing challenge.

Caspases are a family of cysteine proteases that belong to a small clan, the clan CD cysteine proteases. They have a strict requirement for peptides containing an Asp residue at the P_1_ position for substrate cleavage; hence, the name caspase evolved from “cysteinyl aspartate specific protease.” To date, there are 14 caspases identified in humans. Most importantly, caspases are the key-mediators of apoptosis (programmed cell death), and these enzymes play essential roles in inflammation and neurodegeneration^22^^,^^23^. Hence, they have been recognised as novel therapeutic targets for central nervous system diseases in which cell death occurs mainly by an apoptotic mechanism^24^. The roles of caspases in the mechanism of apoptosis has been studied extensively since the mid-1990s^25^^,^^26^. Caspase-3 is a key executioner caspase responsible either partially or totally for proteolytic cleavage of key apoptotic proteins^27^. It functions to decrease or destroy essential homeostatic pathways during the effector phase of apoptosis. Caspase-3 cleaves or activates nuclear enzymes, such as poly(ADP-ribose) polymerase (PARP), the 70 kDa subunit of the U1 small ribonucleoprotein, the catalytic subunit of DNA-dependent protein kinase, and protein kinase Cδ^28^. Compared to the rest of the caspases, caspase-3 is found to be inherently high in abundance in biological environments. In addition, caspase-3 has higher absolute k_cat_/K_m_ values in general^5^. Therefore, most caspase-specific inhibitors will show anti-apoptotic activity via inhibition of caspase-3, also due to high cross-reactivity among caspases themselves. A good caspase inhibitor has to possess a P1 Asp as it is a strict requirement for the caspase family. It is with this motivation, we designed inhibitors specific for caspase-3 with the DEVD sequence. Lately, caspase-6 has been shown to cleave many proteins important in neurodegeneration such as tau, amyloid precursor protein and Huntingtin protein^29^. Hence, an effective caspase-6 inhibitor may offer treatments for neurodegenerative diseases, including Alzheimer’s Disease and Huntington’s Disease.

Legumains are also clan CD cysteine proteases, sharing a common fold with caspases that is unique to this clan. Legumains are acidic lysosomal enzymes that strictly require an asparagine residue at the P_1_ position in order to cleave peptide substrates^30^. Legumain is abundant in human solid tumours and promotes cell migration, tissue invasion and metastasis^31^. The overexpression of legumain in tumour tissues^32^ makes it an attractive target for cancer therapy. Legumain has been described in plants; mammals^33^^,^^34^; the human blood fluke, *Schistosoma mansoni*^35^; the protozoan parasite, *Trichomonas vaginalis*^7^; and the ticks, *Ixodes ricinus*^36^ and *Haemaphysalis longicornis*^31^. In the intestine of *S. mansoni* and *I. ricinus*, legumains contribute to digestion of the blood meal proteins^37^, including haemoglobin, and *trans*-activate other protease zymogens involved in protein digestion^38^. Therefore, in addition to cancer therapy, the selective inhibition of legumains also offers a therapeutic opportunity to effectively treat the diseases caused or transmitted by these parasites^39^ which affect over 200 million people worldwide, with a great majority being located in Africa^40^.

Herein, we show that aza-peptide aldehydes and ketones are selective inhibitors with considerable activity against the proteasome, caspases-3 and -6, as well as the legumains derived from *S. Mansoni* and *I. ricinus*.

## Materials and methods

### Chemistry

All described reactions were carried out in flame/oven-dried glassware under a nitrogen or argon atmosphere, unless otherwise noted. Starting chemical reagents were purchased from reputable suppliers (Sigma Aldrich, St. Louis, MO, USA; Fisher Scientific, Waltham, MA, USA; Matrix Scientific, Columbia, SC, USA; Acros Organics, Fair Lawn, NJ, USA) and used without further purification or purified in accordance with procedures described in *Purification of Common Laboratory Chemicals*. Product purification by flash chromatography was carried out using normal phase 40–64 µm 60 Å silica gel from Sigma Aldrich or using a Teledyne Isco CombiFlash RF + UV auto column system. Dichloromethane, tetrahydrofuran, *N,N*-dimethylformamide, diethyl ether, and pentane solvents were obtained from a solvent purification system (with activated alumina columns). Triethylamine and diisopropylethylamine were freshly distilled over CaH_2_ prior to use and stored under an argon atmosphere. NMR experiments were performed on Bruker 400, 700 or 850 MHz spectrometers. Chemical shifts are expressed in parts per million (δ, ppm) while coupling constant values (J) are given in Hertz (Hz). Residual solvent protons were used as internal standards: for ^1^H NMR spectra CDCl_3_ = 7.26 ppm, DMSO-d_6_ = 2.50 ppm and D_2_O = 4.79 ppm while for ^13^C NMR spectra CDCl_3_ = 77.0 ppm and DMSO-d_6_ = 41.23 ppm; CDCl_3_ and DMSO-d_6_ were purchased from Cambridge Isotope Laboratories. High resolution mass spectra were recorded on a Bruker MicroTOF II instrument with internal sodium formate calibrant under electrospray ionisation (ESI) conditions. Hydrogenations were carried out using a Parr hydrogenation apparatus. Kinetic assays were performed on a Molecular Devices SpectraMax i3.

Abbreviations. The following abbreviations have been used: AMC, 7-amino-4-methyl coumarin; AAla, aza-alanine residue; AAsp, aza-aspartic acid residue; AAsn, aza-asparagine residue; AGly, aza-glycine residue; ALeu, aza-leucine residue; Cbz, Ph-CH_2_-OCO-; Pz, pyrazinyl; DCM, dichloromethane; DMF, *N,N*-dimethylformamide; DMSO, dimethyl sulfoxide; DTT, dithiothreitol; EtOAc, ethyl acetate; *i*BCF, isobutyl chloroformate; MeOH, methanol; NMM, *N*-methylmorpholine; NTA, nitrilotriacetic acid; RT, room temperature; TEV, tobacco etch virus; THF, tetrahydrofuran.

#### Peptide methyl esters


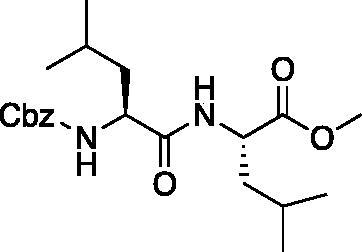


Methyl ((benzyloxy)carbonyl)-*L*-leucyl-*L*-leucinate (Cbz-Leu-Leu-OMe). Cbz-Leu-OH (5.3 g, 20 mmol) was dissolved in dry THF (200 ml) and cooled to −20 °C. NMM (2.18 ml, 20 mmol) and *i*BCF (2.59 ml, 20 mmol) were added dropwise and the mixture was allowed to react for 30 min. Separately, H-Leu-OMe (3.6 g, 20 mmol) was dissolved in dry THF (200 ml) and cooled to −20 °C and NMM (2.18 ml, 20 mmol) was added dropwise. This mixture was allowed to stir for 15 min. The two mixtures were then combined and allowed to stir for 1 h at −20 °C. Following, the solution was allowed to stir while gradually warming to RT for 18 h. The solvent was removed *in vacuo*, and the resulting residue was dissolved with EtOAc, washed with 1 M HCl, H_2_O, saturated NaHCO_3_, and saturated brine. The organic layer was dried over Na_2_SO_4_, and concentrated *in vacuo* to afford the product as a white solid (7.0 g, 90% yield). ^1^H NMR (DMSO-d_6_, 400 MHz): 8.19 (d, *J* = 7.80 Hz, 1H), 7.39–7.28 (m, 5H), 5.00 (s, 2H), 4.33–4.25 (m, 1H), 4.12–4.01 (m, 1H), 3.59 (s, 3H), 1.71–1.36 (m, 6H), 0.93–0.81 (m, 12H).


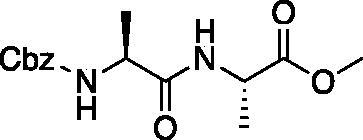


Methyl ((benzyloxy)carbonyl)-*L*-alanyl-*L*-alaninate (Cbz-Ala-Ala-OMe). See procedure and workup of Cbz-Leu-Leu-OMe. A white solid was obtained in 83% yield. ^1^H NMR (DMSO-d_6_, 400 MHz): 8.26 (d, *J* = 7.0 Hz, 1H), 7.40 (d, *J* = 7.6 Hz, 1H), 7.36 (m, 5H), 5.01 (d, *J* = 3.26 Hz, 2H), 4.27 (p, *J* = 7.16 Hz, 1H), 4.08 (p, *J* = 7.35 Hz, 1H), 3.62 (s, 3H), 1.29 (d, *J* = 7.3 Hz, 3H), 1.20 (d, *J* = 7.1 Hz, 3H).


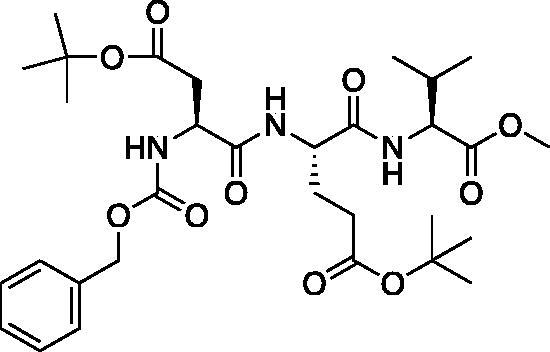


Methyl (5*S*,8*S*,11*S*)-5–(2-(*tert*-butoxy)-2-oxoethyl)-8–(3-(*tert*-butoxy)-3-oxopropyl)-11-isopropyl-3,6,9-trioxo-1-phenyl-2-oxa-4,7,10-triazadodecan-12-oate (Cbz-Asp(O*t*Bu)-Glu(O*t*Bu)-Val-OMe). See procedure and workup of Cbz-Leu-Leu-OMe. A white foam was obtained in 86% yield. ^1^H NMR (DMSO-d_6_, 400 MHz): 8.12 (d, *J* = 7.8 Hz, 1H), 7.92 (d, *J* = 7.8 Hz, 1H), 7.60 (d, *J* = 8.3 Hz, 1H), 7.48–7.23 (m, 5H), 5.04 (d, *J* = 5.7 Hz, 2H), 4.42–4.30 (m, 1H), 4.00–4.19 (m, 2H), 3.62 (s, 3H), 2.65 (dd, *J* = 15.75, 5.66 Hz, 1H), 2.43 (dd, *J* = 16.1, 9.1 Hz, 1H), 2.22 (m, 2H), 2.04 (s, 1H), 1.94–1.81 (m, 1H), 1.79–1.67 (m, 1H), 1.40 (s, 10H, Boc and Val CH), 1.37 (s, 9H), 0.88 (t, *J* = 7.4 Hz, 6H).


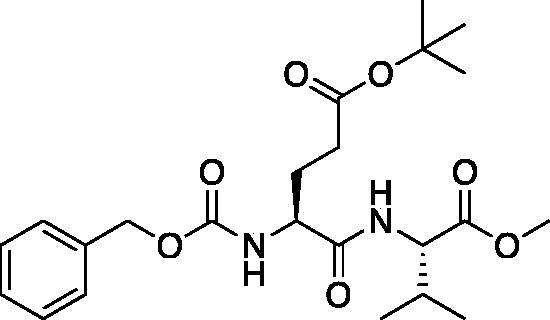


*tert*-Butyl (*S*)-4-(((benzyloxy)carbonyl)amino)-5-(((*S*)-1-methoxy-3-methyl-1-oxobutan-2-yl)amino)-5-oxopentanoate (Cbz-Glu(O*t*Bu)-Val-OMe). See procedure and workup of Cbz-Leu-Leu-OMe. The product was obtained as a colourless oil in quantitative yield. ^1^H NMR (DMSO-d_6_, 400 MHz): 0.88 (t, 6H), 1.39 (s, 9H), 1.72 (m, 1H), 1.85 (m, 1H), 2.05 (q, 1H), 2.25 (t, 2H), 3.63 (s, 3H), 4.16 (m, 2H), 5.02 (d, 2H), 7.35 (m, 5H), 7.42 (d, 1H), 8.10 (d, 1H).


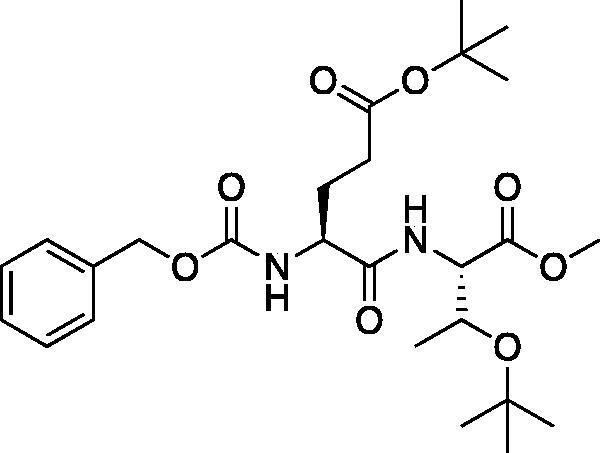


*tert*-Butyl (*S*)-4-(((benzyloxy)carbonyl)amino)-5-(((2*S*,3*S*)-3-(*tert*-butoxy)-1-methoxy-1-oxobutan-2-yl)amino)-5-oxopentanoate (Cbz-Glu(O*t*Bu)-Thr(O*t*Bu)-OMe). At −15 °C a solution of Cbz-Glu(OtBu)-OH (6.74 g, 20 mmol) in THF (200 ml) was treated with NMM (2.97 ml, 20 mmol) and allowed to stir for 10 min. The solution was then treated with iBCF (2.59 ml, 20 mmol) and allowed to stir for 30 min at −15 °C. A second solution of H-Thr(tBu)-OMe (4.51 g, 20 mmol) in THF (200 ml) at −15 °C was treated with NMM (2.97 ml, 20 mmol) and stirred for 15 min. The solutions were combined and stirred for 1 h at −15 °C, then allowed to stir while gradually warming to RT for 16 h. The solvent was removed *in vacuo* and the resulting residue was dissolved in EtOAc, washed with 1 M HCl, H_2_O, saturated Na_2_HCO_3_, and saturated brine. The organic layer was dried over Na_2_SO_4_ and removed *in vacuo* to afford the product as a white solid (8.07 g, 79% yield). ^1^H NMR (DMSO-d_6_, 400 MHz) δ: 7.67 (d, *J* = 8.8 Hz, 1H), 7.53 (d, *J* = 8.2 Hz, 1H), 7.41–7.27 (m, 5H), 5.03 (s, 2H), 4.35 (dd, *J* = 8.8, 2.7 Hz, 1H), 4.24–4.12 (m, 2H), 3.61 (s, 3H), 2.35–2.21 (m, 2H), 1.97–1.83 (m, 1H), 1.78–1.66 (m, 1H), 1.38 (s, 9H), 1.07 (s, 12H); HRMS (ESI) calcd for [C_26_H_40_N_2_O_8_+Na]^+^, 531.2685, found 531.2699 (M + 23).


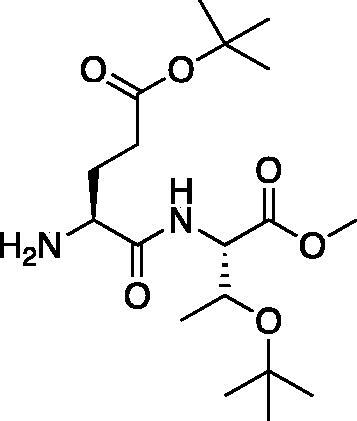


*tert*-Butyl (*S*)-4-amino-5-(((2*S*,3*S*)-3-(*tert*-butoxy)-1-methoxy-1-oxobutan-2-yl)amino)-5-oxopentanoate (H-Glu(O*t*Bu)-Thr(O*t*Bu)-OMe). A solution of *tert*-butyl (*S*)-4-(((benzyloxy)carbonyl)amino)-5-(((2*S*,3*S*)-3-(*tert*-butoxy)-1-methoxy-1-oxobutan-2-yl)amino)-5-oxopentanoate (8.07 g, 15.87 mmol) in MeOH (300 ml) was treated with 10% Pd/C and placed under H_2_ at 40 psi for 2 h. The solution was filtered over Celite and concentrated *in vacuo* to afford the product as a white solid (5.36 g, 90% yield). ^1^H NMR (DMSO-d_6_, 400 MHz) δ: 8.03 (d, *J* = 9.1 Hz, 1H), 4.30 (dd, *J* = 9.0, 2.5 Hz, 1H), 4.16 (qd, *J* = 6.2, 2.6 Hz, 1H), 3.63 (s, 3H), 3.24 (dd, *J* = 7.9, 4.9 Hz, 1H), 3.17 (s, 1H), 2.30 (dd, *J* = 16.7, 8.5 Hz, 2H), 1.98–1.86 (m, 1H), 1.57 (dq, *J* = 13.7, 7.6 Hz, 1H), 1.39 (s, 9H), 1.08 (s, 12H); HRMS (ESI) calcd for [C_18_H_3_4N_2_O_6_ +H]^+^, 375.2495, found: 375.2486 (M + 1).


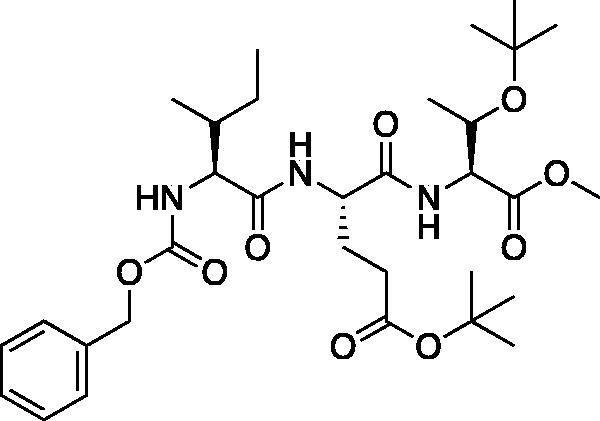


Methyl (5*S*,8*S*,11*S*)-8–(3-(*tert*-butoxy)-3-oxopropyl)-11-((*S*)-1-(*tert*-butoxy)ethyl)-5-((*R*)-*s*-butyl)-3,6,9-trioxo-1-phenyl-2-oxa-4,7,10-triazadodecan-12-oate *tert*-butyl (*S*)-4-(((benzyloxy)carbonyl)amino)-5-(((2*S*,3*S*)-3-(*tert*-butoxy)-1-methoxy-1-oxobutan-2-yl)amino)-5-oxopentanoate (Cbz-Ile-Glu(O*t*Bu)-Thr(O*t*Bu)-OMe). A solution of Cbz-Ile-OH (3.80 g, 14.33 mmol) in THF (100 ml) was cooled to −15 °C followed by the addition NMM (1.58 ml, 14.33 mmol) and allowed to stir for 10 min. The solution was then treated with iBCF (1.86 ml, 14.33 mmol) and allowed to stir for 30 min at −15 °C. Separately, a solution of *tert*-butyl (*S*)-4-amino-5-(((2*S*,3*S*)-3-(*tert*-butoxy)-1-methoxy-1-oxobutan-2-yl)amino)-5-oxopentanoate (5.37 g, 14.33 mmol) in THF (100 ml) at −15 °C was treated with NMM (1.58 ml, 14.33 mmol) and stirred for 15 min. The solutions were combined and allowed to stir for 1 h at −15 °C. Following, the solution was allowed to stir while gradually warming to RT for 16 h. The solvent was removed *in vacuo*, the resulting residue was dissolved in EtOAc, washed with 1 M HCl, H_2_O, saturated Na_2_HCO_3_, and saturated brine. The organic layer was dried over Na_2_SO_4_ and concentrated *in vacuo* to afford the product as a white solid (6.30 g, 10.13 mmol, 70% yield). ^1^H NMR (DMSO-d_6_, 400 MHz) δ: 8.11 (d, *J* = 8.0 Hz, 1H), 7.68 (d, *J* = 8.8 Hz, 1H), 7.41–7.25 (m, 6H, aromatic + NH), 5.03 (s, 2H), 4.52–4.42 (m, 1H), 4.36 (dd, *J* = 8.99, 2.42 Hz, 1H), 4.18–4.11 (m, 1H), 3.93 (t, *J* = 8.51 Hz, 1H), 3.62 (s, 3H), 2.35–2.15 (m, 2H), 1.99–9.80 (m, 1H), 1.79–1.62 (m, 2H), 1.49–1.31 (m, 9H), 1.14–1.02 (m, 14H, CH_3_ + Ile CH_2_), 0.93–0.73 (m, 6H); HRMS (ESI) calcd for [C_32_H_51_N_3_O_9_+Na]^+^, 644.3525, found: 644.3485 (M + 23).

#### Proteasome inhibitors


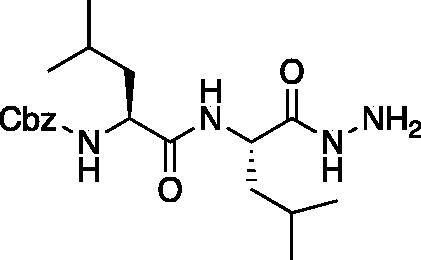


Benzyl ((*S*)-1-(((*S*)-1-hydrazineyl-4-methyl-1-oxopentan-2-yl)amino)-4-methyl-1-oxopentan-2-yl)carbamate (Cbz-Leu-Leu-NHNH_2_). Cbz-Leu-Leu-OMe (7.0 g, 17.8 mmol) was dissolved in MeOH (150 ml) and hydrazine was added dropwise (5.59 ml, 178 mmol). The solution was allowed to stir for 18 h at RT. The solvent was removed *in vacuo* to afford Cbz-Leu-Leu-NHNH_2_ as white solid (7.0 g, 99% yield). ^1^H NMR (DMSO-d_6_, 400 MHz): 9.18 (s, 1H), 7.86 (d, *J* = 9.01 Hz, 1H), 7.44 (d, *J* = 7.71 Hz, 1H), 7.44–7.27 (m, 5H), 5.03 (s, 2H), 4.32–4.23 (m, 1H), 4.10–4.00 (m, 1H), 1.68–1.51 (m, 2H), 1.50–1.28 (m, 4H), 0.82–0.88 (m, 12H).


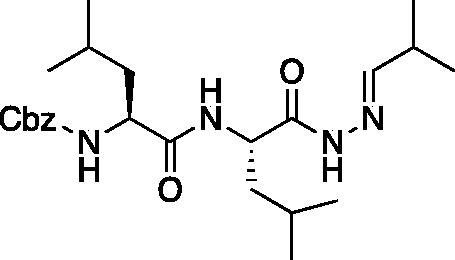


Benzyl ((*S*)-4-methyl-1-(((*S*)-4-methyl-1–(2-((*E*)-2-methylpropylidene)hydrazineyl)-1-oxopentan-2-yl)amino)-1-oxopentan-2-yl)carbamate (Cbz-Leu-Leu-NHN = CHCH(CH_3_)_2_). Cbz-Leu-Leu-NHNH_2_ (7.0 g, 17.8 mmol) was dissolved in dry THF (200 ml) followed by the addition of isobutyraldehyde (1.79 ml, 19.6 mmol) and catalytic acetic acid (1 drop). The solution was allowed to stir for 18 h at RT. The reaction was quenched with saturated NaHCO_3_, extracted with DCM. The organic layer was dried over Na_2_SO_4_ and removed *in vacuo* to afford Cbz-Leu-Leu-NHN = CHCH(CH_3_)_2_ as a white solid (8 g, 99% yield). ^1^H NMR (DMSO-d_6_, 400 MHz): 10.81,11.01 (s, 1H), 7.93, 7.77 (d, *J* = 8.3 Hz, 1H), 7.74 (d, 1H), 7.41 (d, 1H), 7.40–7.24 (m, 5H), 5.02 (s, 2H), 4.12–4.00 (m, 2H), 1.48–1.31 (m, 2H), 1.08–0.99 (m, 1H), 0.93–0.75 (m, 18H).


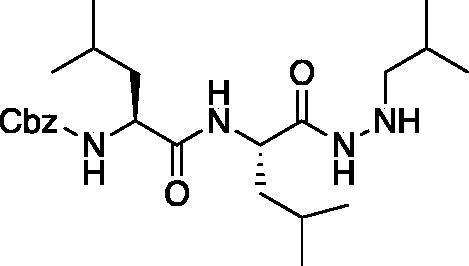


Benzyl ((*S*)-1-(((*S*)-1–(2-isobutylhydrazinyl)-4-methyl-1-oxopentan-2-yl)amino)-4-methyl-1-oxopentan-2-yl)carbamate (Cbz-Leu-Leu-NHNHCHCH(CH_3_)_2_)_._ A solution of benzyl ((*S*)-4-methyl-1-(((*S*)-4-methyl-1–(2-((*E*)-2-methylpropylidene)hydrazinyl)-1-oxopentan-2-yl)amino)-1-oxopentan-2-yl)carbamate (1 g, 2.24 mmol) in MeOH (25 ml) was treated with acetic acid (0.2 ml) followed by sodium cyanoborohydride (170 mg, 2.69 mmol). The reaction was stirred at RT for 16 h, then the pH was adjusted to 10 by addition of 1 M NaOH. The solvent was removed *in vacuo*, and the residue was dissolved in DCM. The organic layer was washed with water, saturated brine, dried over Na_2_SO_4_ and concentrated *in vacuo* to afford the product as a white solid (874 mg, 1.95 mmol, 87% yield). ^1^H NMR (DMSO-d_6_, 400 MHz) δ: 9.25 (d, *J* = 4.2 Hz, 1H), 7.87 (d, *J* = 6.73 Hz, 1H), 7.30 (d, *J* = 7.32, 1H), 7.28–7.39 (m, 5H), 6.47 (d, 1H), 5.10 (s, 2H), 4.33–4.42 (m, 1H), 4.10–4.19 (m, 1H), 2.60 (m, 1H), 1.42–1.82 (m, 9H), 0.8–1.05 (m, 18H). HRMS (ESI) calcd for [C_24_H_40_NO_4+_Na]^+^ 471.2927, Found: 471.2950 (M + 23).


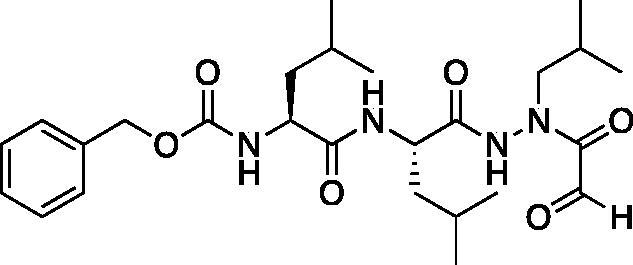


Compound **3**: Benzyl ((*S*)-1-(((*S*)-1–(2-isobutyl-2–(2-oxoacetyl)hydrazinyl)-4-methyl-1-oxopentan-2-yl)amino)-4-methyl-1-oxopentan-2-yl)carbamate. (Cbz-Leu-Leu-ALeu-CHO). A 0 °C solution of benzyl ((*S*)-1-(((*S*)-1–(2-isobutylhydrazinyl)-4-methyl-1-oxopentan-2-yl)amino)-4-methyl-1-oxopentan-2-yl)carbamate (50 mg, 0.11 mmol) in dry DCM (2 ml) was treated with a solution of 2-oxoacetyl chloride (13 mg, 0.14 mmol) in DCM (0.5 ml) followed by DIPEA (0.02 ml, 0.14 mmol). The reaction was warmed to RT and stirred for 2 h. The reaction was quenched with H_2_O and extracted with DCM. The organic layer was washed with saturated NaHCO_3_, dried over Na_2_SO_4_ and concentrated *in vacuo*. The crude product was purified by silica gel chromatography (2% MeOH/DCM) to afford the product as a white solid (18 mg, 32% yield). ^1^H NMR (DMSO-d_6_, 400 MHz) δ 9.08 (s, 1H), 7.96 (s, 1H), 7.49–7.24 (m, 5H), 5.01 (s, 2H), 4.37–4.20 (m, 1 H), 4.14–4.01 (m, 1H), 3.78–3.66 (m, 1H), 1.77–1.32 (m, 7 H), 0.94–0.71 (m, 18H); ^13^C NMR (213 MHz, DMSO-d_6_) δ (ppm) = 189.9, 173.9, 173.0, 157.6, 138.8, 130.2, 129.4, 129.3, 67.0, 54.6, 54.3, 42.5, 42.1, 25.3, 24.2, 23.2, 22.0; HRMS (ESI) calcd for [C_27_H_44_N_4_O_7_+Na]^+^ 559.3108, found 559.3098 (MeOH hemiacetal)


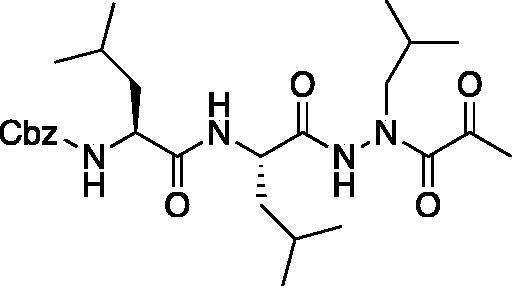


Compound **4**: Benzyl ((*S*)-1-(((*S*)-1–(2-isobutyl-2–(2-oxopropanoyl)hydrazinyl)-4-methyl-1-oxopentan-2-yl)amino)-4-methyl-1-oxopentan-2-yl)carbamate (Cbz-Leu-Leu-ALeu-COMe). A 0 °C solution of pyruvic acid (0.17 ml, 0.69 mmol) in DCM (10 ml) was treated with oxalyl chloride (0.06 ml, 0.72 mmol) followed by DMF (1 drop). The reaction mixture was warmed to RT and stirred 2 h. The solvent was removed *in vacuo*, and residue was dissolved in fresh DCM (5 ml) and chilled to 0 °C. A solution of benzyl ((*S*)-1-(((*S*)-1–(2-isobutylhydrazinyl)-4-methyl-1-oxopentan-2-yl)amino)-4-methyl-1-oxopentan-2-yl)carbamate (200 mg, 0.45 mmol) was added, followed by the addition of DIPEA (0.15 ml, 0.9 mmol). The reaction was stirred 1 h at 0 °C, then warmed to RT and stirred for an additional 16 h. The reaction mixture was diluted with H_2_O and extracted with EtOAc. Organic extracts were dried over Na_2_SO_4_ and concentrated *in vacuo*. The crude product was purified by silica gel chromatography (20–40% EtOAc/Hexanes) to afford the product as a white solid (27 mg, 7.5% yield). ^1^H NMR (DMSO-d_6_, 400 MHz) δ 9.12 (s, 1H), 7.28–7.47 (m, 5H), 6.38 (d, *J* = 7.74 Hz, 1H), 5.24 (br s, 1H), 5.13 (d, *J* = 3.64 Hz, 2H), 4.45 (br s, 1H), 4.05–4.22 (m, 1H), 3.21–3.56 (m, 2H), 2.44 (s, 1H), 1.87–1.98 (m, 1H),1.40–1.75 (m, 6H), 0.86–0.98 (m, 18H).^13^C NMR (213 MHz, DMSO-d_6_): δ (ppm) = 200.3, 174.1, 157.6, 138.8, 130.0, 129.5, 129.3, 67.0, 55.1, 54.5, 51.1, 42.3, 41.7, 28.9, 25.8, 24.7, 24.3, 23.2, 21.5; HRMS (ESI) calcd for [C_27_H_42_N_4_O_6_+Na]^+^ 541.2997, found: 541.2981.


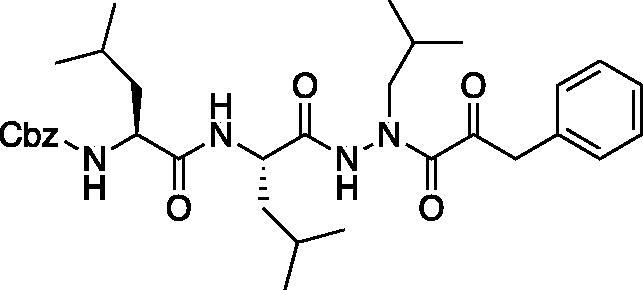


Compound **5**: Benzyl ((*S*)-1-(((*S*)-1–(2-isobutyl-2–(2-oxo-3-phenylpropanoyl)hydrazinyl)-4-methyl-1-oxopentan-2-yl)amino)-4-methyl-1-oxopentan-2-yl)carbamate (Cbz-Leu-Leu-ALeu-COBn). To a solution of phenylpyruvic acid (73 mg, 0.44 mmol) in DCM (2 ml) oxalyl chloride (0.04 ml, 0.44 mmol) was added, followed by DMF (1 drop). The reaction mixture was stirred at RT for 2 h, then concentrated *in vacuo*. The resulting residue was dissolved in fresh DCM (3 ml) and chilled to 0 °C. To this chilled solution was added a solution of benzyl ((*S*)-1-(((*S*)-1–(2-isobutylhydrazinyl)-4-methyl-1-oxopentan-2-yl)amino)-4-methyl-1-oxopentan-2-yl)carbamate (168 mg, 0.37 mmol) in DCM (1 ml) followed by DIPEA (0.13 ml, 0.74 mmol). The reaction was warmed to RT and stirred 16 h. The reaction mixture was diluted with H_2_O and extracted with DCM. The organic layer was dried over Na_s_SO_4_ and concentrated *in vacuo*. The crude product was purified by silica gel chromatography (5% MeOH/DCM) and recrystallised from EtOAc/Et_2_O to afford the pure product as a white solid (38 mg, 17% yield). ^1^H NMR (CDCl_3_, 400 MHz) δ: 8.92 (s, 1H), 7.27–7.41 (m, 10H), 7.15–7.20 (d, *J* = 7.07 Hz, 1H), 6.06 (d, *J* = 9.07 Hz, 1H), 5.04–5.14 (m, 2H), 4.31–4.92 (m, 1H), 3.92–4.20 (m, 3H, CH and Bn), 3.40–3.65 (s, 1H), 3.20–3.37 (m, 1H), 1.84–1.99 (m, 1H), 1.49–1.71 (m, 6H), 1.22–1.35 (m, 2H), 0.81–0.95 (m, 18H). ^13^C NMR (213 MHz, DMSO-d_6_): δ (ppm) = 198.4, 173.9, 156.3, 137.6, 130.4, 130.1, 129.0, 128.9, 128.8, 128.7, 128.2, 128.1, 65.8, 53.9, 53.3, 42.2, 41.1, 24.6, 23.5, 23.3, 22.0, 21.9, 21.1, 20.3; HRMS (ESI) calcd for [C_33_H_46_N_4_O_6_+Na]^+^ 617.3316, found: 617.3308.

#### Caspase-3 inhibitors


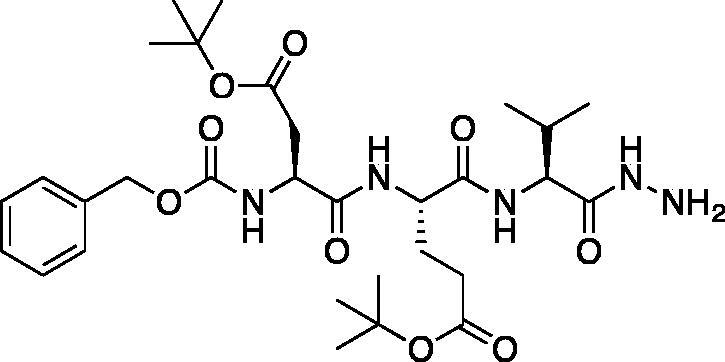


*tert*-Butyl (*S*)-4-((*S*)-2-(((benzyloxy)carbonyl)amino)-4-(*tert*-butoxy)-4-oxobutanamido)-5-(((*S*)-1-hydrazinyl-3-methyl-1-oxobutan-2-yl)amino)-5-oxopentanoate (Cbz-Asp(O*t*Bu)-Glu(O*t*Bu)-Val-NHNH_2_). A solution of methyl (5*S*,8*S*,11*S*)-5–(2-(*tert*-butoxy)-2-oxoethyl)-8–(3-(*tert*-butoxy)-3-oxopropyl)-11-isopropyl-3,6,9-trioxo-1-phenyl-2-oxa-4,7,10-triazadodecan-12-oate (7.9 g, 12.7 mmol) in) MeOH (80 ml) was treated with hydrazine (2.67 ml, 80 mmol). The reaction was stirred at RT for 18 h. The solvent and excess hydrazine were removed *in vacuo* to afford the product as a white solid. This was used for further transformations without additional purification (7.9 g, quantitative). ^1^H NMR (DMSO-d_6_, 400 MHz) δ: 9.16 (s, 1H), 7.98 (d, *J* = 8.1 Hz, 1H), 7.80 (d, *J* = 8.8 Hz, 1H), 7.64 (d, *J* = 8.3 Hz, 1H), 7.44–7.26 (m, 5H), 5.13–4.95 (m, 2H), 4.46–4.11 (m, 4H), 4.04 (t, *J* = 6.83 Hz, 1H), 2.69–2.36 (m, 2H), 2.30–2.14 (m, 2H), 1.96–1.81 (m, 2H), 1.52–1.30 (m, 18H), 0.89–0.76 (m, 6H); HRMS (ESI) calcd for [C_30_H_48_N_5_O_9_]^+^ 622.3447, found: 622.3415.


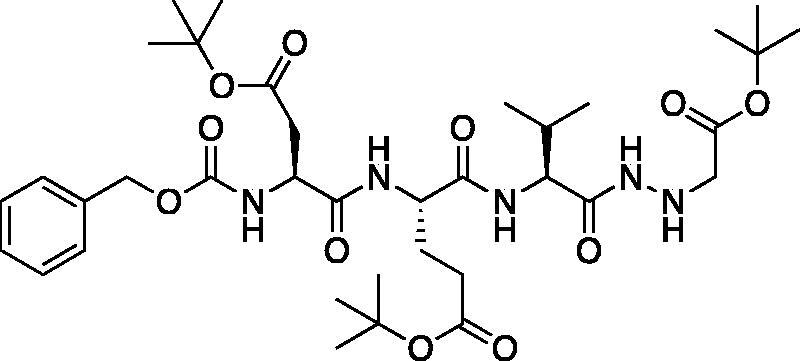


*tert*-Butyl (5*S*,8*S*,11*S*)-5–(2-(*tert*-butoxy)-2-oxoethyl)-8–(3-(*tert*-butoxy)-3-oxopropyl)-11-isopropyl-3,6,9,12-tetraoxo-1-phenyl-2-oxa-4,7,10,13,14-pentaazahexadecan-16-oate (Cbz-Asp(O*t*Bu)-Glu(O*t*Bu)-Val-NHNHCH_2_CO_2_*t*Bu). A −15 °C solution of *tert*-butyl (*S*)-4-((*S*)-2-(((benzyloxy)carbonyl)amino)-4-(*tert*-butoxy)-4-oxobutanamido)-5-(((*S*)-1-hydrazinyl-3-methyl-1-oxobutan-2-yl)amino)-5-oxopentanoate (1 g, 1.6 mmol) in DMF (6 ml) was treated with the dropwise addition of t-butyl bromoacetate (0.26 ml, 1.76 mmol). The reaction was stirred for 30 min, then warmed to RT and stirred for 16 h. The solvent was removed *in vacuo*, and the residue was purified by silica gel chromatography (4% MeOH/DCM) to afford the title product as a white solid (115 mg, 10% yield); ^1^H NMR (DMSO-d_6_, 400 MHz) δ: 9.48 (d, *J* = 6.16 Hz, 1H), 7.95 (d, *J* = 7.44 Hz, 1H), 7.85 (d, *J* = 8.76 Hz, 1H), 7.62 (d, *J* = 8.76 Hz, 1H), 7.47–7.22 (m, 5 H), 5.18–5.11 (m, 1H), 5.10–4.98 (m, 2H), 4.51–4.19 (m, 2H), 4.11–3.99 (m, 1H), 3.41–3.38 (m, 2H), 2.27–2.16 (m, 2H), 1.96–1.69 (m, 5H), 1.46–1.34 (m, 27H), 0.86–0.77 (m, 6H).


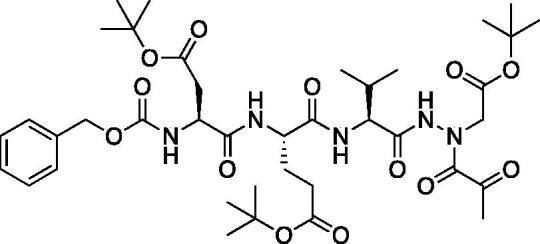
*tert*-Butyl (5*S*,8*S*,11*S*)-5–(2-(*tert*-butoxy)-2-oxoethyl)-8–(3-(*tert*-butoxy)-3-oxopropyl)-11-isopropyl-3,6,9,12-tetraoxo-14–(2-oxopropanoyl)-1-phenyl-2-oxa-4,7,10,13,14-pentaazahexadecan-16-oate (Cbz-Asp(O*t*Bu)-Glu(O*t*Bu)-Val-AAsp(O*t*Bu)-COMe). Prepared following the general procedure for coupling of aza-peptide to pyruvic acid as previously described; (36 mg, 29% yield); ^1^H NMR (CDCl_3_, 400 MHz) δ: 9.02 (s, 1H), 7.90 (d, *J* = 4.99 Hz, 1H), 7.46–7.33 (m, 5H), 6.11–5.99 (m, 1H), 5.22–5.10 (m, 2H), 4.55–4.45 (m, 1H), 4.37–4.25 (m, 2H), 4.24–4.16 (m, 1H), 3.01–2.74 (m, 2H), 2.57–2.39 (m 2H), 2.37 (s, 3H), 2.33–2.24 (m, 1H), 2.19–1.99 (m, 2H), 1.49–1.44 (m, 27H), 0.97–0.92 (m, 6H); HRMS (ESI) calcd for [C_39_H_59_N_5_O_13_+Na]: 828.4002, found: 828.3984.


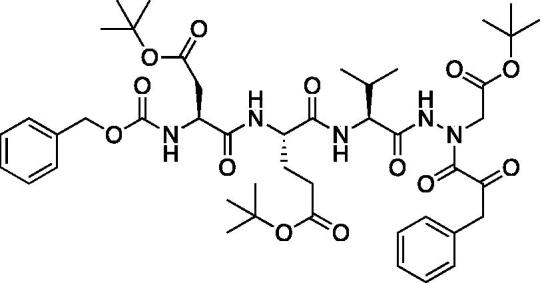
*tert*-Butyl (5*S*,8*S*,11*S*)-5–(2-(*tert*-butoxy)-2-oxoethyl)-8–(3-(*tert*-butoxy)-3-oxopropyl)-11-isopropyl-3,6,9,12-tetraoxo-14–(2-oxo-3-phenylpropanoyl)-1-phenyl-2-oxa-4,7,10,13,14-pentaazahexadecan-16-oate (Cbz-Asp(O*t*Bu)-Glu(O*t*Bu)-Val-AAsp(O*t*Bu)-COBn). This product was prepared following the general procedure for coupling of an aza-peptide to phenylpyruvic acid as previously described: (19 mg, 18% yield); ^1^H NMR (DMSO-d_6_, 400 MHz) δ: 11.07 (s, 1H), 8.00 (d, *J* = 8.3 Hz, 1H), 7.93 (d, *J* = 7.7 Hz, 1H), 7.60 (d, *J* = 8.3 Hz, 1H), 7.40–7.15 (m, 11H), 5.12–4.96 (m, 2H), 4.41–4.28 (m, 2H), 4.21–4.12 (m, 1H), 4.10–3.96 (m, 2H), 2.70–2.57 (m, 1H), 2.47–2.39 (m, 1H), 2.26–2.14 (m, 2H), 1.94–1.80 (m, 2H), 1.77–1.65 (m, 2H), 1.44 (s, 9 H), 1.37–1.32 (m, 18H), 0.81–0.75 (m, 6H); HRMS (ESI) calcd for [C_45_H_63_N_5_O_13_+Na]: 904.4315, found: 904.4282.


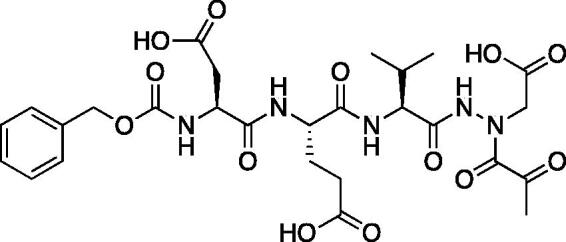


Compound **6**: (5*S*,8*S*,11*S*)-8–(2-Carboxyethyl)-5-(carboxymethyl)-11-isopropyl-3,6,9,12-tetraoxo-14–(2-oxopropanoyl)-1-phenyl-2-oxa-4,7,10,13,14-pentaazahexadecan-16-oic acid (Cbz-Asp-Glu-Val-AAsp-COMe). A solution of *tert*-butyl (5*S*,8*S*,11*S*)-5–(2-(*tert*-butoxy)-2-oxoethyl)-8–(3-(*tert*-butoxy)-3-oxopropyl)-11-isopropyl-3,6,9,12-tetraoxo-14–(2-oxopropanoyl)-1-phenyl-2-oxa-4,7,10,13,14-pentaazahexadecan-16-oate (36 mg, 0.045 mmol) in DCM (0.5 ml) was treated with trifluoroacetic acid (0.5 ml). The reaction was stirred 2 h, then concentrated to dryness *in vacuo* to afford the product as a white solid (27 mg, 95% yield). ^1^H NMR (DMSO-d_6_, 400 MHz) δ: 11.02 (br s, 1H), 8.17–7.78 (m, 2H), 7.73–7.53 (d, *J* = 8.0 Hz, 1H), 7.42–7.29 (m, 5H), 5.04 (s, 2H), 4.42–4.29 (m, 2H), 4.13 (t, *J* = 6.8 Hz, 1H), 2.70–2.57 (m, 2H), 2.48–2.43 (m, 1H), 2.29–2.18 (m, 5H), 1.96–1.82 (m, 2H), 0.82 (d, *J* = 6.4 Hz, 6H). ^13^C NMR (213 MHz, DMSO-d_6_): δ (ppm) = 199.7, 175.7, 173.4, 173.2, 172.9, 172.8, 172.5, 170.4, 170.0, 157.5, 138.6, 130.1, 129.5, 129.4, 67.2, 57.8, 53.3, 53.0, 50.3, 37.9, 31.7, 31.6, 29.1, 20.5, 19.6; HRMS (ESI) calcd for [C_27_H_35_N_5_O_13_+Na]^+^ 660.2124, found 660.2116 (M + 23).


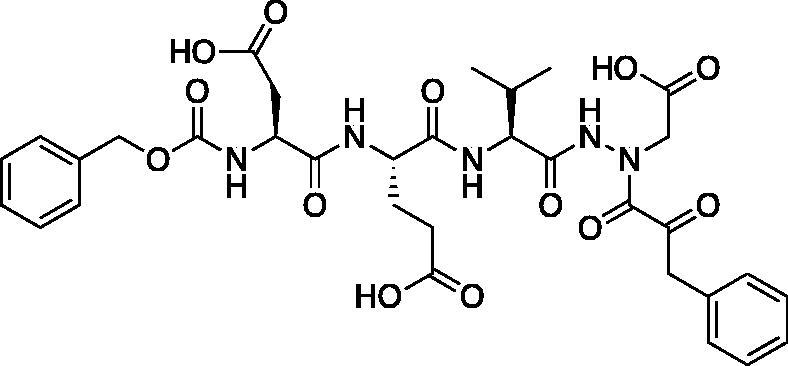


Compound **7**: (5*S*,8*S*,11*S*)-8–(2-Carboxyethyl)-5-(carboxymethyl)-11-isopropyl-3,6,9,12-tetraoxo-14–(2-oxo-3-phenylpropanoyl)-1-phenyl-2-oxa-4,7,10,13,14-pentaazahexadecan-16-oic acid (Cbz-Asp-Glu-Val-AAsp-COBn). *tert*-Butyl (5*S*,8*S*,11*S*)-5–(2-(*tert*-butoxy)-2-oxoethyl)-8–(3-(*tert*-butoxy)-3-oxopropyl)-11-isopropyl-3,6,9,12-tetraoxo-14–(2-oxo-3-phenylpropanoyl)-1-phenyl-2-oxa-4,7,10,13,14-pentaazahexadecan-16-oate (18 mg, 0.02 mmol) was dissolved in a 1:1 DCM/trifluoroacetic acid mixture (1.5 ml). The mixture was stirred for 2.5 h, then solvent was removed, and product was dried *in vacuo* to give the title compound as a white solid (13.6 g, 95%). ^1^H NMR (DMSO-d_6_, 400 MHz) δ: 11.06 (s, 1H), 8.02–7.91 (m, 2H), 7.61 (d, *J* = 8.0 Hz, 1H), 7.42–7.15 (m, 10H), 5.03 (s, 2H), 4.44–4.27 (m, 3H), 4.21–4.12 (m, 2H), 4.06–3.94 (m, 3H), 2.70–2.60 (m, 1H), 2.80–2.18 (m, 2H), 1.95–1.84 (m, 2H), 1.80–1.70 (m, 1H), 0.84–0.70 (m, 6H); ^13^C NMR (213 MHz, DMSO-d_6_): δ (ppm) = 199.3, 173.4, 173.3, 173.0, 172.5, 170.4, 157.5, 138.6, 131.2, 131.7, 131.6, 130.0, 129.9, 129.5, 129.4, 128.6, 67.2, 57.8, 53.4, 53.0, 50.3, 46.8, 37.9, 31.7, 26.4, 20.7, 19.5; HRMS (ESI) calcd for [C_33_H_39_N_5_O_13_+Na]^+^ 736.2437, found: 736.2415 (M + 23).

#### Caspase-6 inhibitor


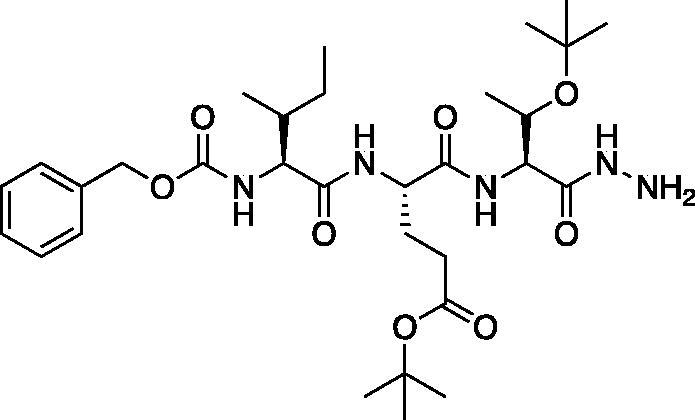
*tert*-Butyl (*S*)-4-((2*S*,3*R*)-2-(((benzyloxy)carbonyl)amino)-3-methylpentanamido)-5-(((2*S*,3*S*)-3-(*tert*-butoxy)-1-hydrazineyl-1-oxobutan-2-yl)amino)-5-oxopentanoate (Cbz-Ile-Glu(O*t*Bu)-Thr(O*t*Bu)-NHNH_2_). A solution of methyl (5*S*,8*S*,11*S*)-8–(3-(*tert*-butoxy)-3-oxopropyl)-11-((*S*)-1-(*tert*-butoxy)ethyl)-5-((*R*)-*s*-butyl)-3,6,9-trioxo-1-phenyl-2-oxa-4,7,10-triazadodecan-12-oate (2.08 g, 3.35 mmol) in MeOH (10 ml) was treated with hydrazine (5.3 ml, 169.8 mmol). The reaction mixture was stirred at RT for 16 h. Excess hydrazine and MeOH were removed *in vacuo* to afford the product as a white solid (9.80 g, 98% yield). ^1^H NMR (DMSO-d_6_, 400 MHz) δ: 8.88 (s, 1H), 8.12 (d, 1H), 7.46 (d, 1H), 7.46–7.35 (m, 6H), 5.02 (s, 2H), 4.38 (m, 1H), 4.25 (s, 2H) 4.15 (m, 1H), 3.40 (m, 2H), 2.30–2.12 (m, 2H), 1.93–1.79 (m, 1H), 1.76–1.63 (m, 2H), 1.13–1.06 (s, 9H), 1.10 (s, 11H), 0.99–0.93 (m, 3H), 0.77–0.82 (m, 6H); HRMS (ESI) calcd for [C_31_H_51_N_5_O_8_+Na]^+^, 644.3638, found: 644.3624 (M + 23).


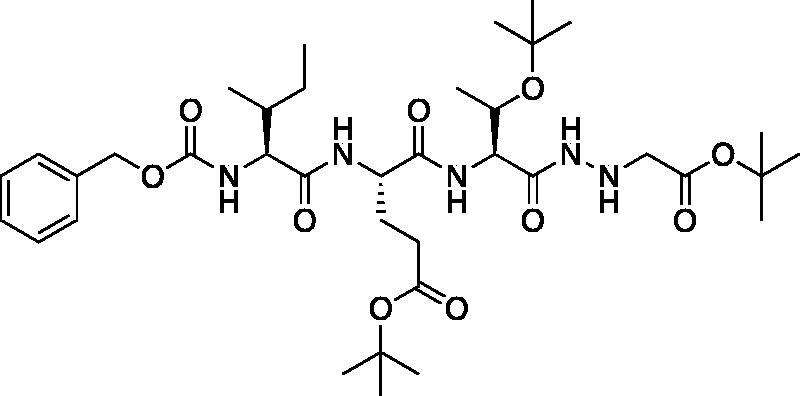
*tert*-Butyl (5*S*,8*S*,11*S*)-8–(3-(*tert*-butoxy)-3-oxopropyl)-11-((*S*)-1-(*tert*-butoxy)ethyl)-5-((*R*)- -butyl)-3,6,9,12-tetraoxo-1-phenyl-2-oxa-4,7,10,13,14-pentaazahexadecan-16-oate (Cbz-Ile-Glu(O*t*Bu)-Thr(O*t*Bu)-NHNHCH_2_CO_2_*t*Bu). A -15 °C solution of *tert*-butyl (*S*)-4-((2*S*,3*R*)-2-(((benzyloxy)carbonyl)amino)-3-methylpentanamido)-5-(((2*S*,3*S*)-3-(*tert*-butoxy)-1-hydrazineyl-1-oxobutan-2-yl)amino)-5-oxopentanoate (1.174 g, 1.89 mmol) in dry DMF (100 ml) was treated with NMM (0.26 ml, 1.89 mmol) and left to stir 15 min. The solution was then treated with t-butyl bromoacetate (0.42 ml, 2.835 mmol) and allowed to stir for 30 min at -15 °C. Following, the solution was allowed to stir while gradually warming to RT for 16 h. The reaction mixture was concentrated *in vacuo* and the crude product was purified by silica gel chromatography (10% MeOH/DCM) to afford the product as a white solid (0.4641 g, 0.6306 mmol, 33% yield). ^1^H NMR (DMSO-d_6_, 400 MHz) δ: 9.14 (d, *J* = 5.8 Hz, 1H), 8.11 (d, *J* = 8.0 Hz, 1H), 7.55 (d, *J* = 8.5 Hz, 1H), 7.36 (m, 6H, aromatic CH + NH), 5.12 (q, *J* = 5.1 Hz, 1H), 5.02 (s, 2H), 4.42–4.31 (m, 1H), 4.18 (dd, *J* = 8.6, 3.7 Hz, 1H), 3.92–3.88 (m, 2H, α CH + CH Thr), 3.38 (s, 2H), 2.36–2.09 (m, 2H), 1.96–1.79 (m, 1H), 1.77–1.58 (m, 2H), 1.47–1.34 (m, 20H, Ile CH_2_ + CH_3_), 1.10 (s, 9H), 1.00–0.93 (m, 3H), 0.90–0.74 (m, 6H); HRMS (ESI) calcd for [C37H61N5O10 +Na]^+^ 758.4318, found 758.4301 (M + 23).


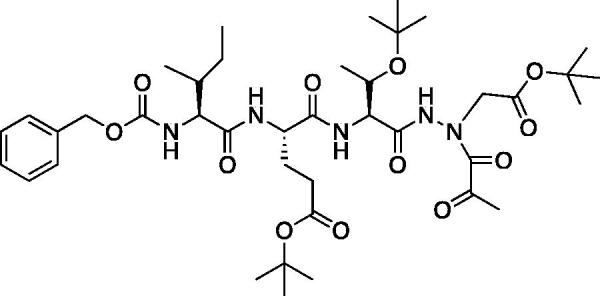
*tert*-Butyl (5*S*,8*S*,11*S*)-8–(3-(*tert*-butoxy)-3-oxopropyl)-11-((*S*)-1-(*tert*-butoxy)ethyl)-5-((*R*)-*s*-butyl)-3,6,9,12-tetraoxo-14–(2-oxopropanoyl)-1-phenyl-2-oxa-4,7,10,13,14-pentaazahexadecan-16-oate (Cbz-Ile-Glu(O*t*Bu)-Thr(O*t*Bu)-AAsp(O*t*Bu)-COMe). A solution of pyruvic acid (0.02 ml, 0.20 mmol) in dry DCM (50 ml) was treated with oxalyl chloride (0.02 ml, 0.217 mmol) followed by 1 drop of DMF (cat). The reaction was stirred at RT for 3 h, then concentrated *in vacuo*. The resulting residue was dissolved in dry DCM (50 ml), chilled to 0 °C and treated dropwise with a solution of *tert*-butyl (5*S*,8*S*,11*S*)-8–(3-(*tert*-butoxy)-3-oxopropyl)-11-((*S*)-1-(*tert*-butoxy)ethyl)-5-((*R*)-*s*-butyl)-3,6,9,12-tetraoxo-1-phenyl-2-oxa-4,7,10,13,14-pentaazahexadecan-16-oate (100 mg, 0.136 mmol) in dry DCM (20 ml) and DIPEA (0.05 ml, 0.272 mmol). The reaction was stirred for 16 h at RT. The reaction was quenched with H_2_O, and washed with saturated brine. The organic layer was dried over Na_2_SO_4_ and concentrated *in vacuo* The crude product was purified by silica gel chromatography (10% MeOH/DCM) to afford product as a white solid (22.8 mg, 21% yield). ^1^H NMR (DMSO-d_6_, 400 MHz) δ: 10.77 (s, 1H), 8.05 (d, *J* = 7.9 Hz, 1H), 7.75 (d, *J* = 7.8 Hz, 1H), 7.42–7.24 (m, 6H, aromatic CH + NH), 5.02 (s, 2H), 4.54–4.44 (m, 1H), 4.27 (dd, *J* = 8.0, 4.5 Hz, 1H), 3.92 (t, *J* = 8.1 Hz, 1H), 3.95–3.89 (m, 1H), 2.23 (s, 5H), 1.85 (m, 1H), 1.70 (m, 2H), 1.88–1.39 (m, 18H), 1.11 (s, 11H), 0.95 (m, 3H), 0.86–0.74 (m, 6H); HRMS (ESI) calcd for [C_40_H_63_N_5_O_12_+Na]^+^ 828.4373, found 828.4360 (M + 23).


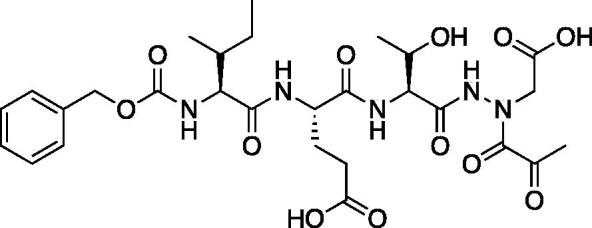


Compound **8**: (5*S*,8*S*,11*S*)-5-((*R*)-*s*-butyl)-8–(2-carboxyethyl)-11-((*S*)-1-hydroxyethyl)-3,6,9,12-tetraoxo-14–(2-oxopropanoyl)-1-phenyl-2-oxa-4,7,10,13,14-pentaazahexadecan-16-oic acid (Cbz-Ile-Glu-Thr-AAsp-COMe). *tert*-butyl (5*S*,8*S*,11*S*)-8–(3-(*tert*-butoxy)-3-oxopropyl)-11-((*S*)-1-(*tert*-butoxy)ethyl)-5-((*R*)-*s*-butyl)-3,6,9,12-tetraoxo-14–(2-oxopropanoyl)-1-phenyl-2-oxa-4,7,10,13,14-pentaazahexadecan-16-oate (22.8 mg, 0.028 mmol) was dissolved in 1:1 DCM/TFA (20 ml) at 0 °C and allowed to stir 30 min the warmed to RT and stirred an additional 1 h. The solution was concentrated *in vacuo* to afford the product as a white solid (15.8 mg, 85% yield). ^1^H NMR (DMSO-d_6_, 400 MHz) δ: 10.82 (s, 1H), 8.00 (d, *J* = 7.99 Hz, 1H), 7.80 (d, *J* = 7.96 Hz, 1H), 7.35 (m, 6H), 5.02 (s, 2H), 4.89 (m, 1H), 4.39 (m, 1H), 4.17 (m, 1H), 3.92 (m, 2H), 2.24 (s, 3H), 1.90 (m, 1H), 1.70 (m, 2H), 1.39 (m, 2H), 1.22 (s, 2H), 0.99 (m, 3H), 0.80 (m, 6H); ^13^C NMR (175 MHz, DMSO-d_6_) δ (ppm) = 201.4, 178.6, 176.2, 172.2, 170.1, 167, 163, 149.1, 145.3, 135.9, 130.0, 129.9, 129.4, 67.1, 65.3, 62.3, 60.8, 57.4, 36.6, 31.6, 25.9, 21.2, 18.2, 12.7, 11.8. HRMS (ESI) calcd for [C_28_H_39_N_5_O_12_+Na]^+^ 660.2487, found 660.2485 (M + 23).

#### Legumain inhibitors


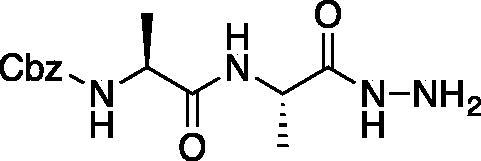


Benzyl ((*S*)-1-(((*S*)-1-hydrazinyl-1-oxopropan-2-yl)amino)-1-oxopropan-2-yl)carbamate (Cbz-Ala-Ala-NHNH_2_). To a solution of methyl ((benzyloxy)carbonyl)-*L*-alanyl-*L*-alaninate (Cbz-Ala-Ala-OMe) (2.0 g, 6.4 mmol) in MeOH (50 ml), hydrazine (2.12 ml, 64 mmol) was added dropwise. The reaction was allowed to stir at RT for 18 h. MeOH and excess hydrazine were removed *in vacuo* and the resulting white solid was washed with EtOAc to afford a white solid (1.68 g, 5.4 mmol, 85% yield). ^1^H NMR (DMSO-d_6_, 400 MHz) δ 9.05 (s, 1H), 7.90 (d, *J* = 7.7 Hz, 1H), 7.42 (d, *J* = 7.6 Hz, 1H), 7.41–7.26 (m, 5H), 4.97–5.07 (m, 2H), 4.20–4.26 (m, 2H), 4.04–4.11 (m, 2H), 1.19 (s, 3 H), 1.18 (s, 3H).


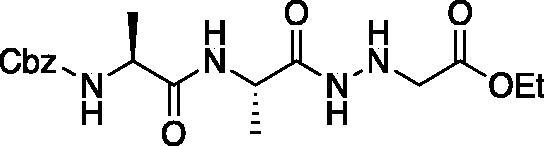


Ethyl ((*S*)-2-((*S*)-2-(((benzyloxy)carbonyl)amino)propanamido)propanamido)glycinate (Cbz-Ala-Ala-NHNHCH_2_CO_2_Et). To solution of benzyl ((*S*)-1-(((*S*)-1-hydrazinyl-1-oxopropan-2-yl)amino)-1-oxopropan-2-yl)carbamate (1.68 g, 5.4 mmol) and NMM (0.65 ml, 5.9 mmol) in DMF (8 ml) at −10 °C ethyl bromoacetate (0.65 ml, 5.9 mmol) was added dropwise. The reaction was stirred at −10 °C for 30 min, then warmed to RT and stirred for an additional 16 h. The reaction was then concentrated *in vacuo*, and the resulting residue was purified by silica gel chromatography (1:9 MeOH/DCM) to afford a white solid (375 mg, 20% yield). ^1^H NMR: (DMSO-d_6_, 400 MHz) δ 9.34 (d, *J* = 5.8 Hz, 1H), 7.92 (d, *J* = 7.58 Hz, 1H), 7.41–7.47 (d, *J* = 7.7 Hz, 1H), 7.28–7.41 (m, 5H), 5.18 (q, *J* = 4.94 Hz, 1H), 5.02 (s, 2H), 4.18–4.26 (m, 1H), 4.01–4.14 (m, 2H), 3.48 (d, *J* = 5.24 Hz, 2H), 1.35–1.44 (m, 3H), 1.15–1.22 (m, 6H).


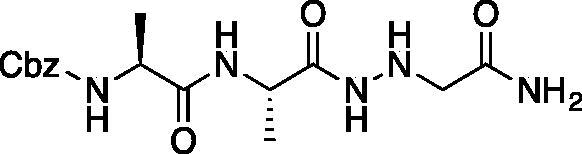


Benzyl ((*S*)-1-(((*S*)-1–(2-(2-amino-2-oxoethyl)hydrazinyl)-1-oxopropan-2-yl)amino)-1-oxopropan-2-yl)carbamate (Cbz-Ala-Ala-NHNHCH_2_CONH_2_). A 7 M solution of ammonia in MeOH (13.5 ml, 94.5 mmol) was added to a 0 °C solution of ethyl ((*S*)-2-((*S*)-2-(((benzyloxy)carbonyl)amino)propanamido)propanamido)glycinate (375 mg, 0.95 mmol) in DMF (0.4 ml). NaCN (5 mg, 0.095 mmol) was added, and the reaction vessel was sealed. The mixture was stirred at 0 °C for 2 h, then warmed to RT and stirred for 48 h. The reaction was concentrated *in vacuo* and the product was precipitated with 9:1 DCM/MeOH to afford a white solid (343 mg, 98% yield). ^1^H NMR (DMSO-d_6_, 400 MHz) δ 9.33 (s, 1H), 7.98 (d, *J* = 7.0 Hz, 1H), 7.30–7.47 (m, 5H), 7.12 (s, 1H), 5.22 (t, *J* = 4.13 Hz, 1H), 4.97–5.08 (m, 2H), 4.15–4.24 (m, 1H), 4.01–4.11 (m, 1H), 3.20 (d, *J* = 5.23 Hz, 2H), 1.20 (s, 3H), 1.18 (s, 3H).


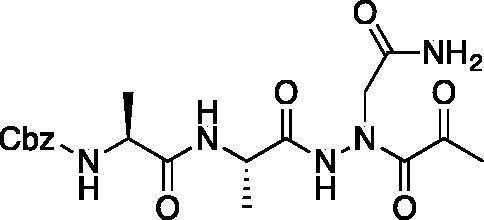


Compound **10**: Benzyl ((*S*)-1-(((*S*)-1–(2–(2-amino-2-oxoethyl)-2-(2-oxopropanoyl)hydrazinyl)-1-oxopropan-2-yl)amino)-1-oxopropan-2-yl)carbamate (Cbz-Ala-Ala-AAsn-COMe). A 0 °C of pyruvic acid (0.15 ml, 0.62 mmol) in DCM (10 ml) was treated with oxalyl chloride (0.06 ml, 0.66 mmol), followed by one drop of DMF (cat). The reaction was stirred at 0 °C for 30 min, then warmed to RT and stirred an additional 1.5 h. To this mixture was added DIPEA (0.14 ml, 0.82 mmol) followed by a solution of benzyl ((*S*)-1-(((*S*)-1–(2-(2-amino-2-oxoethyl)hydrazinyl)-1-oxopropan-2-yl)amino)-1-oxopropan-2-yl)carbamate (150 mg, 0.41 mmol) in 1:2 DCM/DMF (1 ml). The reaction was stirred for 16 h, then concentrated *in vacuo* and purified by silica chromatography (1:9 MeOH/DCM) to afford a white solid (24 mg, 13% yield). ^1^H NMR (DMSO-d_6_, 400 MHz) δ: 10.41 (s, 1H), 9.05 (s, 1H), 8.11–8.00 (m, 1 H), 7.55–7.26 (m, 7H), 5.03 (s, 2H), 4.13–4.05 (m, 2H), 3.18 (d, *J* = 5.24 Hz, 2H), 2.03–1.98 (m, 3H), 1.55–1.42 (m, 6 H); HRMS (ESI) calcd for [C_19_H_25_N_5_O_7+_Na]^+^ 458.1652, Found: 458.1634 (M + 23).


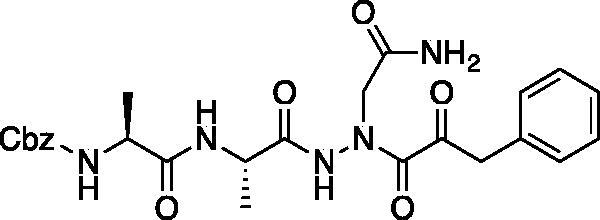


Compound **11**: Benzyl ((*S*)-1-(((*S*)-1–(2–(2-amino-2-oxoethyl)-2-(2-oxo-3-phenylpropanoyl)hydrazinyl)-1-oxopropan-2-yl)amino)-1-oxopropan-2-yl)carbamate (Cbz-Ala-Ala-AAsn-COBn). A solution of phenylpyruvic acid (172 mg, 1.05 mmol) in DCM (3 ml) was treated with oxalyl chloride (0.09 ml, 1.05 mmol), followed by DMF (1 drop), and the reaction was stirred for 1.5 h at RT, then concentrated *in vacuo*. The resulting residue was taken up in fresh DCM (5 ml). To this solution was added a solution of benzyl ((*S*)-1-(((*S*)-1–(2-(2-amino-2-oxoethyl)hydrazinyl)-1-oxopropan-2-yl)amino)-1-oxopropan-2-yl)carbamate (80 mg, 0.21 mmol) in DCM (1.5 ml), followed by DIPEA (0.07 ml, 0.21 mmol). The reaction was stirred at RT for 16 h, then concentrated *in vacuo* and purified by silica gel chromatography (2–10% MeOH/DCM) then recrystallised from EtOAc/Et_2_O to afford the product as a white solid (19 mg, 0.037 mmol, 18% yield). ^1^H NMR (DMSO-d_6_, 400 MHz) δ: 8.13 (d, *J* = 7.89 Hz, 1H), 7.17–7.40 (m, 12H, ArH and NH2), 4.94–5.08 (m, 2H), 4.17–4.31 (m, 1H), 4.05–4.13 (m, 1H), 4.00 (s, 2H), 1.05–1.30 (m, 6H); ^13^C NMR (175 MHz, DMSO-d_6_) δ (ppm) = 197.0, 168.4, 161.8, 157.4, 130.1, 130.0, 129.97, 129.94, 129.9, 129.5, 129.4, 67.0, 60.0, 59.5, 51.5, 45.9, 45.5, 19.8**;** HRMS (ESI) calcd for [C_25_H_29_N_5_O_7+_Na]^+^ 534.1965, Found: 534.1975 (M + 23).

#### Proteasome kinetic assay

Human 20S proteasome, 700 KDa, was purchased from Boston Biochem (50 µg, 2 µM (1.4 mg/mL)) in 50 mM HEPES buffer (pH 7.6, 100 mM NaCl, 1 mM DTT). Assay buffer was prepared as follows: 20 mM HEPES buffer pH 7.8, 0.5 mM EDTA, 0.037% SDS. Proteasome specific substrate Suc-LLVY-AMC was purchased from Boston Biochem and used as a fluorogenic substrate; λ_ex_ = 380 nm, λ_em_ = 442 nm. 18 µL of 2 µM proteasome solution was diluted with 425 µL H_2_O to make a 0.06 µM stock enzyme solution for kinetic assays. In a 96-well plate suitable for fluorometric assays was added 86 µL assay buffer, 2 µL inhibitor, 2 µL enzyme substrate, and 10 µL enzyme stock solution (0.06 nM enzyme concentration in well). Fluorescence at 442 nM was monitored for 10 min. The enzyme activity was measured in triplicate at varying concentrations of inhibitor (0, 25, 50, 100 µM in DMSO) and substrate (10, 20, 50, 100 µM in DMSO for K_i_ measurements, and 100 µM for IC_50_ measurements) by converting the slope of the plot of fluorescence intensity at 442 nm vs time for time points between 4 and 8 min using Beer-Lambert’s law. IC_50_ values were obtained from non-linear fitting of the data to a competitive inhibition model using GraphPad Prism 7.0 software. The proteasome β5 chymotrypsin-like active site activity assay was performed as previously described^41^.

#### Caspase kinetic assay

Caspase-3 and caspase-6 were generous gifts from Prof. Guy Salvesen’s laboratory and then used at Ohio State University. Caspase activities were measured in 100 mM HEPES, 10% sucrose, 0.1% CHAPS, 10 mM DTT, pH 7.5 at 37 °C. To 5,000 µL of this buffer, 100 µL of freshly prepared 1 M DTT and 4,900 µL DI H_2_O were added resulting in the caspase assay buffer. Studies were performed at 37 °C.

#### Caspase-3

Caspase-3 specific substrate Ac-DEVD-AMC was purchased from Cayman Chemical Company and used as fluorogenic substrate; λ_ex_ = 353 nm, λ_em_ = 442 nm. 2 µL of 33 µM caspase-3 solution was diluted with 4998 µL caspase assay buffer to make a 13.2 nM enzyme stock solution. In a 96-well plate suitable for fluorometric assays, 80 µL assay buffer, 5 µL inhibitor, 5 µL enzyme substrate (0.1 mM), and 10 µL enzyme stock solution were added. Fluorescence at 442 nM was monitored for 10 min. The enzyme activity was measured in triplicate at varying concentrations of inhibitor (0, 25, 50, 100 µM in DMSO) and substrate (0.1 mM in DMSO) by converting the slope of the plot of fluorescence intensity at 442 nm vs time for the time points between 4 and 8 min using Beer-Lambert’s law. IC_50_ values were obtained from non-linear fitting of the data to a competitive inhibition model using GraphPad Prism 7.0 software.

#### Caspase-6

Caspase-6 specific substrate Ac-VEID-AFC was purchased from ChemCruz and used as a fluorogenic substrate; λ_ex_ = 400 nm, λ_em_ = 505 nm. To 45 µL of assay buffer, 5 µL of the 19.7 µM enzyme was added. This resulting enzyme solution was further diluted 1:5 with assay buffer resulting in a 0.32 µM solution. In a 96-well plate suitable for fluorometric assays was added 80 µL assay buffer, 5 µL inhibitor, 5 µL enzyme substrate (0.1 mM), and 10 µL enzyme stock solution (32 nM enzyme concentration in well). The enzyme activity was measured in triplicate at varying concentrations of inhibitor (0, 25, 50, 100 µM in DMSO) and substrate (0.1 mM in DMSO) by converting the slope of the plot of fluorescence intensity at 442 nm vs time for the time points between 4 and 8 min using Lambert-Beer’s law. IC_50_ values were obtained from non-linear fitting of the data to a competitive inhibition model using GraphPad Prism 7.0 software.

#### Legumain kinetic assay

*Schistosoma mansoni* and *Ixodes ricinus* legumain zymogens were expressed in the yeast, *Pichia pastoris*, as described^36^^,^^42^. Legumain kinetic assay details: zymogens were activated for 3 h (*Ir*) or overnight (*Sm*) at room temperature in 0.1 M Na-Ac pH 4.0; 5 mM DTT. Activities were measured in 0.1 M Na-Ac pH 5.5; 2.5 mM DTT, 0.1% PEG 6000 buffer. The substrate used was Cbz-AAN-AMC with 50 µM final concentration in the assay. Inhibitors were prepared in DMSO (1% DMSO in activity assay). Measurements were taken with an incubation period of 10 min at room temperature, and the enzyme activity was measured in triplicate. Asparaginyl Endopeptidase (AE) activity assay was performed as previously described^7^.

#### Cathepsin B kinetic assay

Human Liver Cathepsin B (purified) was purchased from Enzo Life Sciences (in 50 mM sodium acetate, pH 5.0 containing 1 mM EDTA). The Cathepsin B activity assay is performed in 0.1 M phosphate, 1.25 mM EDTA, 0.01% Brij, pH 6.0 buffer at 37 °C. Cathepsin B specific substrate Cbz-Arg-Arg-AMC was purchased from Sigma Aldrich and used as fluorogenic substrate; λ_ex_ = 380 nm, λ_em_ = 442 nm. The assay buffer was prepared as follows 0.1 M K_3_PO_4_, 1.25 mM EDTA, 0.01% Brij 35 with a pH of 6 was made with 1 mM DTT added immediately prior to assay. To 100 µL of assay buffer, 5 µL of the received 0.427 mg/mL Cathepsin B solution was added. The resulting solution was further diluted 1:6 of Cathepsin B to assay buffer for a final concentration of 2.9 µg/mL. In a 96-well plate suitable for fluorometric assays was added 85 µL assay buffer, 5 µL inhibitor, 5 µL enzyme substrate, and 5 µL enzyme stock solution (0.145 µg/mL enzyme concentration in well). The enzyme activity was measured in triplicate at varying concentrations of inhibitor (0, 62.5, 125 µM in DMSO) and substrate (0.1 mM in DMSO) by converting the slope of the plot of fluorescence intensity at 442 nm vs time for the time points between 4 and 8 min using Lambert-Beer’s law. IC_50_ values were obtained from non-linear fitting of the data to a competitive inhibition model using GraphPad Prism 7.0 software.

#### *α*-Chymotrypsin kinetic assay

α-Chymotrypsin from bovine pancreas, type II lyophilised powder, >40 units/mg protein activity was purchased from Sigma Aldrich and was tested in 50 mM sodium phosphate, pH 7.5 buffer at 37 °C. To prepare the enzyme stock solution, 1.6 mg α-chymotrypsin was dissolved in 1,600 µL assay buffer. The resulting stock enzyme solution was further diluted 1:200 stock to assay buffer for a final stock concentration of 5 µg/mL. α-Chymotrypsin specific substrate Suc-LLVY-AMC was purchased from Boston Biochem and used as a fluorogenic substrate; λ_ex_ = 353 nm, λ_em_ = 442 nm. In a 96-well plate suitable for fluorometric assays was added 80 µL assay buffer, 5 µL inhibitor, 5 µL enzyme substrate, and 10 µL enzyme stock solution (0.5 µg/mL enzyme concentration in well). The enzyme activity was measured in triplicate at varying concentrations of inhibitor (0, 50, 125 µM in DMSO) and substrate (0.1 mM in DMSO), by converting the slope of the plot of fluorescence intensity at 442 nm vs time for the time points between 4 and 8 min using Lambert-Beer’s law. IC_50_ values were obtained from non-linear fitting of the data to a competitive inhibition model using GraphPad Prism 7.0 software.

### Protein expression and purification

The expression and purification of caspase-3 was performed as previously described^43^^,^^44^ with several modifications. The cDNA encoding the caspase-3 sequence with the addition of an N-terminal 6xHis-tag followed by a TEV protease recognition sequence was codon optimised for expression in *E. coli* (GenScript) and placed in a pET15b vector (Invitrogen). The protein was expressed in *E. coli* at 30 °C for 24 h after 200 mM IPTG induction. Cells were pelleted by centrifugation and lysed by sonication. The lysate was cleared by centrifugation and purified by Ni-NTA (GE Healthcare) and Q-Sepharose (GE Healthcare) chromatography. TEV protease (1.25 mg) was added to the purified protein during overnight dialysis at 4 °C and the TEV protease and free His-tag were removed by reverse Ni-NTA purification. Any remaining impurities were removed and the buffer was exchanged by size-exclusion chromatography on a Superdex 200 Increase column equilibrated with 20 mM Tris, 20 mM NaCl, 10 mM DTT, pH = 7.5 yielding protein with purity >95% by SDS-PAGE. The protein was concentrated to 5 mg/mL by ultrafiltration (10 kDa MWCO, EMD Millipore), flash frozen, and stored at −80 °C until ready to use.

### Crystallisation and structure determination

The protein was inhibited by adding 20 mM Cbz-DEVaD-COMe inhibitor in DMSO to a final concentration of 2.1 mM giving a final concentration of 4 mg/ml caspase-3/inhibitor complex. Protein crystallisation was performed by hanging-drop vapour diffusion at 298 K with 1:1 ratio of mother liquor (50 mM sodium citrate, 15.33% PEG 6000, 7.5% glycerol, 10 mM DTT, pH = 6.2) to the protein/inhibitor complex. Crystals were observed within one day and reached their maximum dimensions within 7 days. Crystals were cryoprotected in a 1:4 solution of glycerol:mother liquor and frozen on the instrument in the cryo-stream at 100 K. X-ray diffraction data were collected in-house on a Compact HomeLab X-ray diffractometer (Rigaku) with MicroMax 003i generator, AFC-11 4-axis goniometer, and Pilatus 200 K detector.

Diffraction images were indexed, integrated, and scaled with HKL3000 (HKL Research, Inc.) in the P22_1_2_1_ space group with a maximum resolution of 2.73 Å. Phases were determined by molecular replacement with 2H5I^43^ as a model using Phaser-MR in the Phenix software package^45^. Coordinates and restraints for the sulphur-bound ligand were created using eLBOW^46^ and PRODRG (GlycoBioChem Ltd.) and modified with REEL^47^. Refinement was performed using phenix.refine^48^ in the Phenix software package and all coordinates and electron density maps were visualised using winCOOT^49^. Statistical information for the solved structure can be viewed in Supplemental information.

## Results and discussion

### Chemical synthesis

The general preparation of aza-peptide aldehydes and ketones involves the synthesis of the substituted hydrazide precursor^5^ and its coupling to oxoacetyl chloride (prepared *in situ* with thionyl chloride) for aldehydes and to methyl or benzyl pyruvic acid chlorides for ketones, respectively.

**Figure 2.**
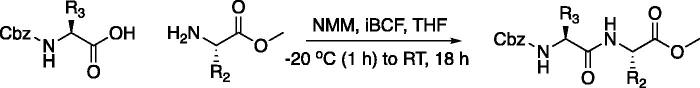
General peptide coupling approach.

The tripeptidyl inhibitor synthesis begins with an isobutyl chloroformate mediated coupling between the methyl ester of the P_2_ amino acid and the carboxybenzyl protected P_3_ amino acid (Figure 2).

**Figure 3.**
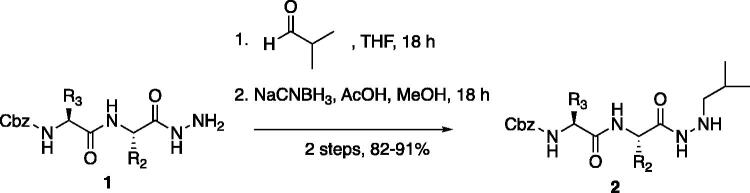
Substituted hydrazide precursor synthesis.

From the P_3_-P_2_ dipeptide, the P_1_ aza-amino acid is added by, first, the addition of excess hydrazine to the methyl ester to furnish the corresponding hydrazide **1** in quantitative yield (Figure 3), followed by reductive amination with an appropriate aldehyde to install the P_1_ side chain. The aza-leucine derivative was synthesised by a two-step reductive amination, first reacting the hydrazide Cbz-Leu-Leu-NHNH_2_ and isobutyraldehyde to afford a hydrazide intermediate, which was subsequently reduced with sodium cyanoborohydride to effectively install the isobutyl side chain on the hydrazide to form the aza-leucine intermediate **2** in good yield (Figure 3).

**Figure 4.**
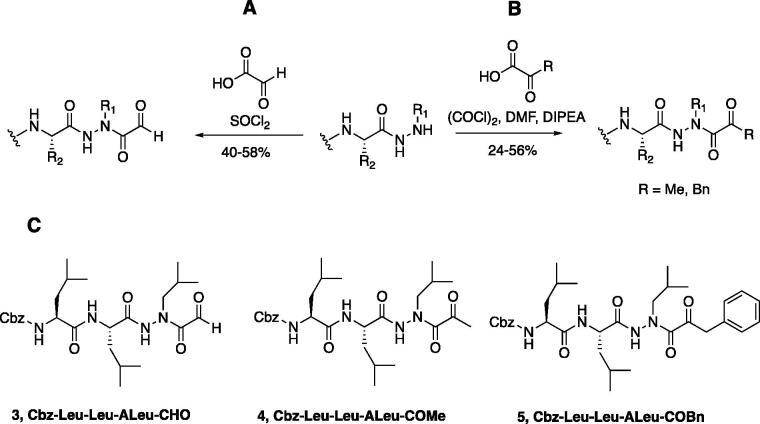
(A) Coupling procedure for aza-peptide aldehyde warhead. (B) Coupling procedure for aza-peptide ketone warhead. (C) Library of synthesised aza-peptide aldehyde and ketone inhibitors for the proteasome.

With the synthesis of the inhibitor backbones complete, the final step was the coupling of the aza-peptide intermediate to the electrophilic carbonyl warheads (Figure 4). The aza-peptide aldehyde inhibitor was prepared by conversion of glyoxylic acid to the corresponding acid chloride by refluxing in thionyl chloride, and then coupling this intermediate to the aza-peptide hydrazides in a manner analogous to the synthesis of the ketone inhibitors (Figure 4(A)).

Initially, we proposed a methyl and a benzyl ketone to explore the tolerance of functional groups in the S_1_’ pocket where the ketone R’ group was anticipated to reside. We have chosen the methyl and benzyl groups for two reasons. (a) The methyl group is the smallest group possible for our proposed aza-peptide ketone design, and (b) the phenyl group, if tolerated, can be derivatised further to make more specific interactions at the prime site. Coupling of the warheads was performed by conversion of readily available pyruvic acid or phenylpyruvic acid to the corresponding acid chloride intermediates via reaction with oxalyl chloride, and then acyl substitution of the acid chloride with the aza-peptide hydrazide intermediates, thereby yielding the final aza-peptide ketones (Figure 4(B)). These two described methods were used for the synthesis of the aza-peptide aldehyde **3** as well as ketones **4** and **5**, as shown in Figure 4(C).

**Figure 5.**
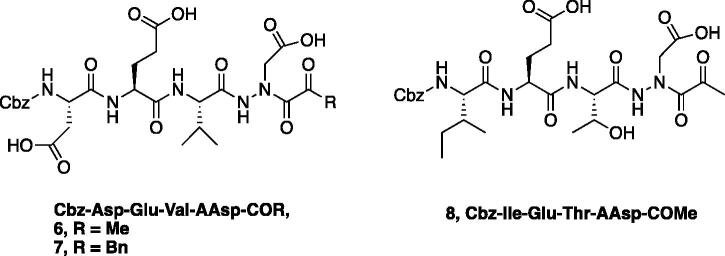
Aza-peptide ketone inhibitors designed for caspase-3 and -6.

A series of caspase-3 aza-peptide ketone inhibitors as well as one caspase-6 aza-peptide ketone bearing a P_1_ aza-aspartate residue were synthesised and evaluated (Figure 5).

**Figure 6.**
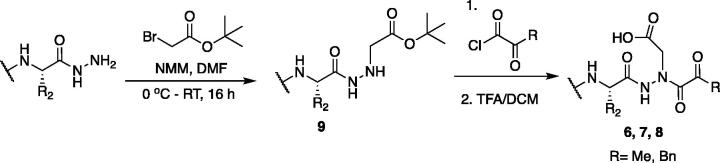
Synthesis of the aza-aspartate caspase inhibitors.

The tri-peptide hydrazides Z-Asp(O*^t^*Bu)-Glu(O*^t^*Bu)-Val-NHNH_2_ and Z-Ile-Glu(O*^t^*Bu)-Thr(O*^t^*Bu)-NHNH_2_ were synthesised using the general peptide coupling and hydrazide formation conditions described for the proteasome inhibitors above. The aza-aspartate residue was synthesised according to Figure 6. Nucleophilic substitution of the hydrazide with *tert*-butyl bromoacetate gave the *tert*-butyl aspartate protected intermediates of the general structure **9**. Analogous warhead coupling methods to those described in Figure 4 were then used, followed by a trifluoroacetic acid deprotection of the *tert*-butyl groups, affording the final inhibitors **6**, **7**, and **8**.

**Figure 7.**
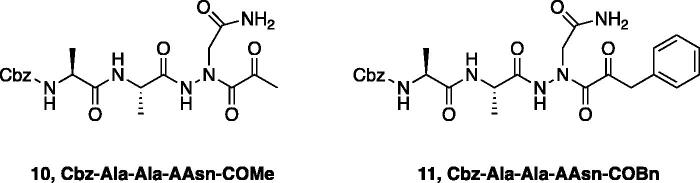
Aza-peptide ketone inhibitors designed for the clan CD legumain protease.

Inhibitors were also designed and synthesised to target the legumain protease. The clan CD cysteine protease, legumain, has a preference to cleave substrates after an asparagine amino acid residue and has the optimal substrate sequence Cbz-Ala-Ala-Asn^50^. The aza-peptide ketone inhibitors **10** and **11** depicted in Figure 7, targeting inhibition of legumain, were synthesised.

**Figure 8.**
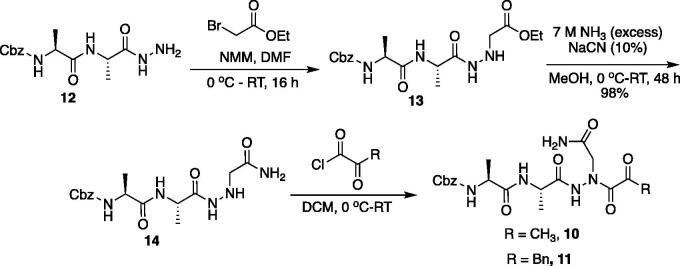
Synthesis of aza-asparagine legumain inhibitors.

The synthesis of legumain inhibitors **10** and **11** was performed by adaptation of the previous syntheses. The alanine-alanine di-peptide hydrazide **12** intermediate (Figure 8) was synthesised using an isobutyl chloroformate peptide coupling method, followed by the reaction of the resulting methyl ester with hydrazine. The intermediate **12** was subjected to reaction with ethyl bromoacetate to afford the alkylated hydrazide **13**. Amidation of the ethyl ester was carried out with ammonia in a sealed pressure tube to afford **14**, as previously reported^5^. These precursors were subsequently coupled with the methyl or benzyl keto-warhead to give the final inhibitors **10** and **11**.

### *In vitro* inhibition

We synthesised a total of eight final compounds. Each compound was characterised by ^1^H and ^13^C nuclear magnetic resonance spectra and electrospray ionisation mass spectra (see the Materials and Methods section). The results of the inhibition of the various aza-peptide aldehydes and ketones are summarised in Tables 1–3.

**Table 1.** Inhibition of β5 active site of the human 20S proteasome by aza-peptide aldehydes and ketones

Compound	Structure	IC_50_ (μM)
3	Cbz-Leu-Leu-ALeu-CHO	9.02 ± 1.82
4	Cbz-Leu-Leu-ALeu-COMe	14.56 ± 2.39
5	Cbz-Leu-Leu-ALeu-COBn	10.11 ± 4.49
MG132	Cbz-Leu-Leu-Leu-CHO	0.0142 ± 0.003^a^

^a^This is a K_i_ value.

**Table 2.** Inhibition of human caspase-3 and caspase-6 by aza-peptide ketones

Compound	Structure	IC_50_ (μM)	
		Caspase-3	Caspase-6
6	Cbz-Asp-Glu-Val-AAsp-COMe	7.74 ± 1.88	51.93 ± 10.64
7	Cbz-Asp-Glu-Val-AAsp-COBn	13.36 ± 4.61	64.23 ± 45.40
8	Cbz-Ile-Glu-Thr-AAsp-COMe	122 ± 83.31	9.08 ± 3.02

**Table 3.** Inhibition of *S. mansoni* and *I. ricinus* legumains by aza-peptide ketones

Compound	Structure	IC_50_ (μM)	
		*S. mansoni*	*I. ricinus*
10	Cbz-Ala-Ala- AAsn-COMe	>100	>100
11	Cbz-Ala-Ala- AAsn-COBn	22.23 ± 4.21	17.82 ± 11.17

Compounds **3**, **4** and **5** are designed to target the β5, chymotrypsin-like (CT-L) active site of the human 20S proteasome, and thus are based on the ideal tripeptidyl sequence Leu-Leu-Leu^51^. To our knowledge, this is the first study where a chemical electrophilic warhead bearing an aza-P1 residue was designed and tested with the proteasome. Compounds **3**, **4** and **5** showed inhibition in the mid-µM range, suggesting that the proteasome active site can actually tolerate the aza-modification at the P_1_ position (Table 1). Inhibition rates by the aldehyde **3** and ketones **4** and **5** seemed fairly close with the aldehyde **3** being slightly more potent with an IC_50_ value of 9.02 µM. The benzyl group of the ketone inhibitor **5** was well accommodated at the prime site for this warhead motif with an IC_50_ value of 10.11 µM, allowing further derivatisation on the aromatic ring for more potency and specificity. The overall performance of our compounds, particularly compound **3**, can be directly compared to the commercially available aldehyde inhibitor MG132 (Cbz-Leu-Leu-Leu-CHO). MG132 is a very potent, but non-selective, proteasome inhibitor. In our assay, we have determined the K_i_ value of MG132 of the β5, chymotrypsin-like (CT-L) active site of the 20S proteasome as 14.28 ± 3.06 nM. This value compares well with the previously reported K_i_ values of MG132 as 2–4 nM^52^, “few nanomolar”^53^, and of the close analog MG115 (Cbz-Leu-Leu-Nle-CHO) as 21 nM^54^ using the same substrate Suc-LLVY-AMC as in our assay.

Compounds **6** and **7** were designed as tetrapeptides to target human caspase-3 with the ideal sequence Asp-Glu-Val-Asp and compound **8** targets human caspase-6 with its optimal sequence Ile-Glu-Thr-Asp as caspases require a minimum of four amino acids in their recognition sequence^26^. Compounds **6**, **7**, and **8** show low-to-mid µM range inhibition with their target enzyme, where the best inhibitor is ketone **6** with an IC_50_ value of 7.74 µM against caspase-3 (Table 2). As expected, the caspase-3 specific compounds **6** and **7** show less activity against caspase-6; however, due to the strict P_1_ Asp requirement for cleavage for all caspases, it is a challenge to obtain highly selective inhibition among different caspase family members. Likewise, compound **8** inhibits caspase-6 with an IC_50_ value of 9.08 µM, 13 times more effectively than it inhibits caspase-3, emphasising the importance of the preference in caspase-3 for the P4 Asp residue. Within caspase-3 and -6, the benzyl compound **7** demonstrates the lowest inhibition for both enzymes, with IC_50_ values of 13.36 and 64.23 µM, respectively.

Compounds **10** and **11** were tested for their ability to inhibit the legumain proteases *S. mansoni* and *I. ricinus* and displayed IC_50_ values in the mid-µM range (Table 3). Compound **11** inhibited the *I. ricinus* and *S. mansoni* legumains with IC_50_ values of 17.82 µM and 22.23 µM, respectively, suggesting a favourable aromatic interaction on the prime site for both enzymes. The aza-asparagine methyl ketone **10** inhibitor did not show inhibition towards either of the legumains at the highest concentration tested (100 µM).

In addition to screening all of the compounds with their respective target enzymes, we also tested them for possible cross-reactivities with human liver cathepsin B and bovine pancreas α-chymotrypsin (Table 4). Cathepsin B was chosen to represent the clan CA type of cysteine proteases, and α-chymotrypsin representing the general chymotrypsin fold of serine proteases. The literature comparison compound, MG132 potently inhibits calpains and various lysosomal cathepsins with K_i_ values of 5–12 nM^54^ in addition to the proteasome^52^. Our compounds, on the other hand, showed no inhibition of either cathepsin B or α-chymotrypsin at concentrations 50 µM and higher, suggesting no *in vitro* cross-reactivity and high selectivity among different families of proteases (see the Materials and Methods section for more details). While *in vitro* and *in vivo* selectivities do not always correlate, initial observation of *in vitro* selectivity is valuable information, and then, one can interpret subsequent *in vivo* behaviour in light of *in vitro* data more precisely. The initial *in vitro* selectivity is a desired and promising asset to these compounds, rendering their further development into potential drug candidates both compelling and exciting.

**Table 4.** Cross-reactivity of human cathepsin B and bovine pancreas α-chymotrypsin with aza-peptide aldehydes and ketones

Compound	Structure	Inhibition (μM)	
		Cathepsin B	α-Chymotrypsin
3	Cbz-Leu-Leu-ALeu-CHO	NI^a^	NI^a^
4	Cbz-Leu-Leu-ALeu-COMe	NI^a^	NI^a^
5	Cbz-Leu-Leu-ALeu-COBn	ND	ND
6	Cbz-Asp-Glu-Val-AAsp-COMe	NI^b^	NI^b^
7	Cbz-Asp-Glu-Val-AAsp-COBn	NI^b^	NI^b^
8	Cbz-Ile-Glu-Thr-AAsp-COMe	NI^c^	NI^c^
10	Cbz-Ala-Ala-AAsn-COMe	ND	NI^b^
11	Cbz-Ala-Ala-AAsn-COBn	ND	ND

NI: no inhibition; ND: not determined; NI up to ^a^62.5 μM, ^b^50 μM, ^c^125 μM.

### Binding mode and mechanism of inhibition

**Figure 9.**
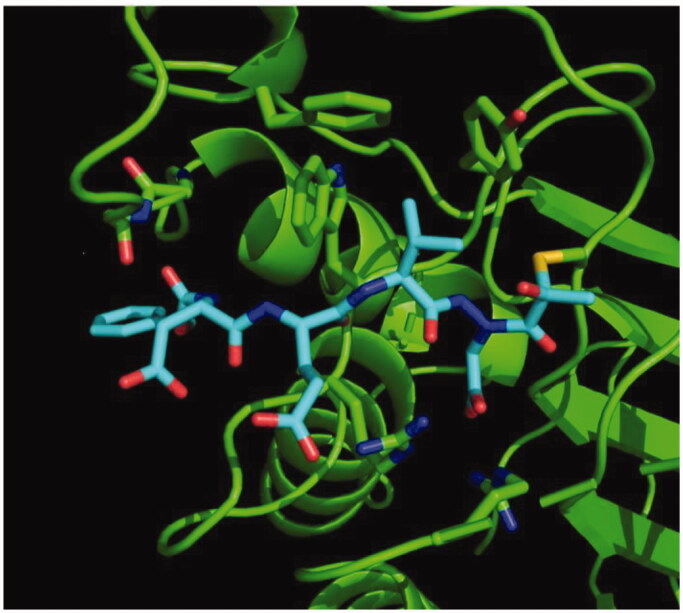
Caspase-3 in complex with Cbz-Asp-Glu-Val-AAsp-COMe (Compound **6**). Compound **6** is observed residing in the active site of caspase-3 at a resolution of 2.73 Å after thiohemiacetal covalent-bond formation to the methyl ketone warhead of **6**.

We determined the X-ray crystal structure of caspase-3 in complex with our aza-peptide methyl ketone inhibitor **6**, Cbz-Asp-Glu-Val-AAsp-COMe (Figure 9).

**Figure 10.**
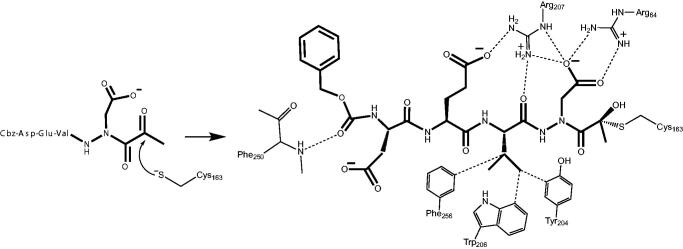
Mechanism of inhibition of caspase-3 by the aza-peptide methyl ketone inhibitor Cbz-Asp-Glu-Val-AAsp-COMe (Compound **6**). The inhibitor ketone carbonyl carbon is the site of nucleophilic addition by the active-site Cys163 sulphur atom, resulting in covalent bond formation.

We observed a similar binding mode at the active site in accordance with the previously determined X-ray structures of caspase-3 with inhibitors such as Ac-Asp-Glu-Val-Asp-H^55^ and Ac-Asp-Val-Ala-Asp-FMK^56^. In all of these structures, key hydrogen-bonding interactions between the P1 Asp side chain of the inhibitor with two Arg side chains (Arg64 and Arg207 in our structure) on the protein backbone is the determinant of the strict P1 Asp specificity. We also observed that the P3 Glu forms a hydrogen bond with Arg207 as well. In addition, the P2 Val side chain is well accommodated by the Phe256, Trp206 and Tyr204 aromatic rings. Most importantly, we observed the nucleophilic addition of the active site Cys163 of caspase-3 at the carbonyl carbon resulting in a tetrahedral thiohemiacetal adduct (Figure 10).

**Figure 11.**
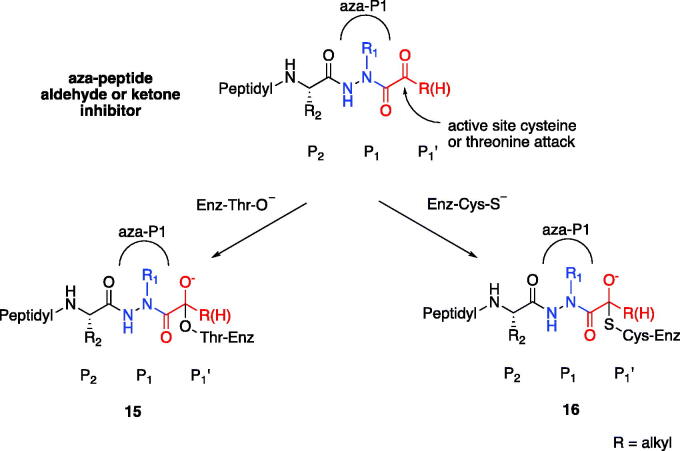
Proposed mechanism of inhibition by aza-peptide aldehydes and ketones reacting with (a) the proteasome and with (b) other clan CD cysteine proteases. The carbonyl carbon is expected to be the site of attack by nucleophilic residues: a Thr-O with the proteasome and a Cys-S with clan CD cysteine proteases.

Based on our observations from the X-ray crystal structure of caspase-3 in complex with compound **6**, we propose that the inhibition mechanism by the proteasome proceeds via a nucleophilic attack of the deprotonated Thr-O^–^ at the carbonyl carbon of our warhead, thereby forming a reversible tetrahedral hemiacetal adduct **15** as shown in Figure 11. Similarly, we propose that inhibition by the other clan CD cysteine proteases, such as the legumains, occurs by nucleophilic addition of the deprotonated active site Cys-S^–^, similarly forming the reversible tetrahedral thiohemiacetal adduct **16**.

## Conclusions

In summary, we have developed a new class of peptidyl protease inhibitors bearing an aza-P1 residue that are effective against the proteasome, caspases and legumains. The inhibitors were designed and synthesised based on their ideal substrate sequence. The tripeptide Leu-Leu-Aza-Leu sequence was chosen to target the β5, chymotrypsin-like active site of the proteasome. Likewise, Asp-Glu-Val-Aza-Asp, Ile-Glu-Thr-Aza-Asp and Ala-Ala-Aza-Asn were chosen as preferred substrate sequences for the caspases-3, and -6 and legumains, respectively.

Eight novel compounds were synthesised and evaluated for their ability to inhibit their respective target proteases *in vitro*, and were shown to be low-to-mid-µM range inhibitors. Each inhibitor was also tested for cross-reactivity with cathepsin B and α-chymotrypsin, and resulted in no inhibition (>50 µM). X-ray crystallographic data with caspase-3 revealed a tetrahedral adduct with compound **6**, providing insight into the mechanism of inhibition. The micromolar range of inhibitory potency for these aza-peptide aldehydes and ketones could be attributed to the fact that the point of the nucleophilic attack is one chemical bond away towards the C-terminus from where the scissile bond normally would reside (Figure 1). However, it is also advantageous that the proteasome, caspases-3, and -6, and legumains still tolerate this aza-P1 design, whereas the classical cysteine protease cathepsin B and α-chymotrypsin do not. These differences provide an opportunity for tuneability as well as selectivity, and possibly less off-target reactivity as observed for bortezomib, carfilzomib and ixazomib.

We hypothesise that the potency of aza-peptide ketones can be improved by increasing the inductive effect on the prime site’s alkyl group which will render the ketone carbonyl to be more electrophilic. Our future, second generation compounds will be designed and synthesised accordingly. Overall, the *in vitro* selectivity and reversible nature of these inhibitors makes them more desirable drug candidates, as any possible cross reactivity would be reversible, arguably resulting in less severe side effects.

## Supplementary Material

Supplemental Material
