# Supplementary material for: Design, synthesis, and in vitro evaluation of aza-peptide aldehydes and ketones as novel and selective protease inhibitors
Source: J Enzyme Inhib Med Chem. 2020 Jul 7;35(1):1387–402. doi: 10.1080/14756366.2020.1781107 (PMC7470110; doi:10.1080/14756366.2020.1781107)
Supplement: Supplemental Material [file IENZ_A_1781107_SM3693.pdf]

Supplemental Information

Design, Synthesis, and *In Vitro* Evaluation of Aza-peptide Aldehydes and Ketones as Novel and Selective Protease Inhibitors

Thomas S. Corrigan,[a] Leilani M. Lotti Diaz,[a] Sarah E. Border,[a] Steven C. Ratigan,[e] Kayla Q. Kasper,[a] Daniel Sojka,[b] Pavla Fajtova,[c] Conor R. Caffrey,[c] Guy S. Salvesen,[d] Craig A. McElroy,[e] Christopher M. Hadad,[a] Özlem Doğan Ekici*[a,f]

[a]*Department of Chemistry and Biochemistry, The Ohio State University, Columbus, Ohio 43210*

[b]*Institute of Parasitology, Biology Centre of the Czech Academy of Sciences, CZ-370 05 Ceske Budejovice, Czech Republic*

[c]*Center for Discovery and Innovation in Parasitic Diseases, Skaggs School of Pharmacy and Pharmaceutical Sciences, University of California San Diego, La Jolla, California 92093*

[d]*Sanford Burnham Prebys Medical Discovery Institute, La Jolla, California 92037*

[e]*Division of Medicinal Chemistry and Pharmacognosy, College of Pharmacy, The Ohio State University, Columbus, Ohio 43210*

[f]*Department of Chemistry and Biochemistry, The Ohio State University at Newark, Newark, Ohio 43055*

Corresponding Author

* Özlem Doğan Ekici

Email: dogan-ekici.1@osu.edu

Department of Chemistry and Biochemistry, The Ohio State University at Newark, Newark, Ohio 43055

Statistical information for the solved Caspase-3/Cbz-DEVaD-COMe structure can be viewed in Table 1.

Table 1. Data collection and refinement statistics.

Wavelength	1.54178
Resolution range	27.6 - 2.732 (2.83 - 2.732)
Space group	P 2 21 21
Unit cell	43.864 66.86 97.862 90 90 90
Total reflections	24884
Unique reflections	7558 (487)
Multiplicity	3.3
Completeness (%)	93.46 (61.65)
Mean I/sigma(I)	4.5
Wilson B-factor	44.48
R-merge	0.174
R-meas	0.282
R-pim	0.106
CC1/2	0.862 (0.472)
Reflections used in refinement	7557 (487)
Reflections used for R-free	755 (49)
R-work	0.2196 (0.3335)
R-free	0.2582 (0.3801)
Total number of non-hydrogen atoms	2058
Protein non-hydrogen atoms	1883
Ligand non-hydrogen atoms	46
Protein residues	237
RMS(bonds)	0.004
RMS(angles)	1.06
Ramachandran favored (%)	96.12
Ramachandran allowed (%)	3.45
Ramachandran outliers (%)	0.43

Rotamer outliers (%)	2.48
Clashscore	3.97
Average B-factor	39.84
macromolecules	39.65
ligands	49.53

Statistics for the highest-resolution shell are shown in parentheses.
